# Unraveling ice multiplication in winter orographic clouds via in-situ observations, remote sensing and modeling

**DOI:** 10.1038/s41612-024-00671-9

**Published:** 2024-06-22

**Authors:** Paraskevi Georgakaki, Anne-Claire Billault-Roux, Romanos Foskinis, Kunfeng Gao, Georgia Sotiropoulou, Maria Gini, Satoshi Takahama, Konstantinos Eleftheriadis, Alexandros Papayannis, Alexis Berne, Athanasios Nenes

**Affiliations:** 1 Laboratory of Atmospheric Processes and their Impacts (LAPI), School of Architecture, Civil & Environmental Engineering, Ecole Polytechnique Fédérale de Lausanne, Lausanne, Switzerland; 2 Environmental Remote Sensing Laboratory (LTE), School of Architecture, Civil & Environmental Engineering, Ecole Polytechnique Fédérale de Lausanne, Lausanne, Switzerland; 3 Laser Remote Sensing Unit (LRSU), Physics Department, National Technical University of Athens, Zografou, Greece; 4 Center for Studies of Air Quality and Climate Change, Institute of Chemical Engineering Sciences, Foundation for Research and Technology Hellas, Patras, Greece; 5 Environmental Radioactivity & Aerosol technology for atmospheric & Climate impacT Lab (ENRACT), Institute of Nuclear and Radiological Sciences and Technology, Energy and Safety, National Centre of Scientific Research “Demokritos”, Ag. Paraskevi, Greece; 6 Division of Environmental Physics and Meteorology, Department of Physics, University of Athens, Athens, Greece

**Keywords:** Atmospheric science, Climate sciences

## Abstract

Recent years have shown that secondary ice production (SIP) is ubiquitous, affecting all clouds from polar to tropical regions. SIP is not described well in models and may explain biases in warm mixed-phase cloud ice content and structure. Through modeling constrained by in-situ observations and its synergy with radar we show that SIP in orographic clouds exert a profound impact on the vertical distribution of hydrometeors and precipitation, especially in seeder-feeder cloud configurations. The mesoscale model simulations coupled with a radar simulator strongly support that enhanced aggregation and SIP through ice-ice collisions contribute to observed spectral bimodalities, skewing the Doppler spectra toward the slower-falling side at temperatures within the dendritic growth layer, ranging from −20 °C to −10 °C. This unique signature provides an opportunity to infer long-term SIP occurrences from the global cloud radar data archive, particularly for this underexplored temperature regime.

## Introduction

The distribution of ice and liquid water within mixed-phase clouds (MPCs) significantly affects surface cloud radiative forcing^1,2^ and the hydrological cycle^3,4^. MPCs exhibit spatial heterogeneity at spatial scales lower than 100 m, with spatially separated ice- and liquid-phase clusters^5,6^. This heterogeneity impacts the efficiency of the Wegener-Bergeron-Findeisen (WBF) process^7–9^ (where ice crystals grow at the expense of cloud droplets) and the rate of cloud glaciation. Accurately representing these processes in numerical weather prediction (NWP) and climate models remains a major challenge and a source of model bias^10–12^.

Ice crystal number concentration (ICNC) is a key microphysical parameter for MPCs and can be modulated by the availability of ice nucleating particles (INPs)^13,14^. The sparsity of INPs^15^ at temperatures above −20 °C cannot account for observed ICNCs in MPCs. Secondary ice production (SIP) following the initial primary ice nucleation events must be considered to bridge the gap between the limited availability of INPs and the abundance of ICNCs^16,17^. Atmospheric models neglecting the effect of SIP are therefore prone to underestimate simulated ICNCs at warm subzero temperatures with important implications for their radiative properties and microphysical evolution^18,19^.

The importance of SIP has been widely acknowledged in laboratory^20,21^, field^22–24^, remote sensing^25–28^, and modeling studies^29–31^ worldwide^32^. The most commonly invoked SIP processes include the Hallett-Mossop (HM) or rime-splintering process^33,34^, ice-ice collisional break-up (BR)^35,36^, and droplet-shattering (DS) during freezing^37,38^. While HM is routinely included in atmospheric models, its efficiency is limited to a narrow temperature range of −8 °C to −3 °C and specific cloud microphysical configurations. Recent experimental studies even suggest potential overestimation of the efficiency of this process^39^. Vigorous convective downdrafts^40,41^ and associated subsaturated regions may also foster the break-up of graupel and dendritic snow particles from sublimation (SUBBR)^42,43^.

A major challenge is the ability to detect the presence of SIP in global MPCs, ideally with insights on its intensity and mechanisms. Without such information, models lack a key microphysical constraint that impedes progress in the description of MPCs. Ground-based remote sensing observations of clouds can provide key information for constraining SIP^25,27^. One approach is to use lidar and radar retrievals to extract ice multiplication factors (i.e., ICNCs/INPs); application of such a method in wintertime orographic MPCs indicated the widespread occurrence of SIP^44^. Doppler spectrograms from vertically-pointing radars provide another powerful approach, as they often exhibit multimodal distributions within the temperature range associated with SIP or within the dendritic growth layer (DGL), typically between −20 °C and −10 °C^45^. These distributions suggest interactions between fast-falling and slower-falling particles within the radar volume from riming^46^ or new ice formation^25,27,47,48^. Significant ambiguity however remains on the interpretation of these signals, as downdrafts, horizontal winds, turbulence or other measurement uncertainties can affect their interpretation.

Multi-frequency, polarimetric radar measurements, whether obtained from scanning^49,50^ or profiling cloud radars^47,51^, have extensively contributed to discerning signatures of the HM mechanism, primarily associated with the production of columnar ice crystals within the narrow HM temperature zone. While valuable for assessing HM representation in NWP models^52^, there has been limited exploration of signatures related to SIP at colder subzero temperatures, particularly in combining modeling with forward radar simulators to evaluate the robustness of SIP mechanisms identified from radar characteristics. Radar-based studies suggest the potential influence of alternative SIP processes, like BR or DS, generating disk-like particles^25^ and possibly skewing the Doppler spectra towards the slower-falling hydrometeor population^45^. Here we use an NWP model with advanced SIP descriptions coupled with a forward radar simulator^53–55^ to interpret vertically-pointing cloud radar observations and deduce the presence of SIP without the need for polarimetric measurements. Identifying characteristic fingerprints associated with specific microphysical processes, including SIP, not only in Doppler spectrograms but also in associated higher-order radar moments, holds significant implications for systematically leveraging the abundant cloud radar data for larger-scale, statistical applications.

## Results

We employed the mesoscale Weather Research and Forecasting model (WRF) coupled with an updated version of the Morrison^56^ microphysics scheme (hereafter denoted as M09), which incorporates detailed descriptions of SIP processes, to investigate the microphysics driving an intense snowfall event observed on December 18, 2021 in mainland Greece. Doppler spectrograms along with timeseries of radar moments were captured by a W-band spectral zenith profiler (WProf)^57^, deployed at Mount Helmos in the Peloponnese (Greece), as part of the Cloud-AerosoL InteractionS in the Helmos background TropOsphere (CALISHTO) campaign (https://calishto.panacea-ri.gr/). The radar observations provided valuable insights into the snowfall microphysics, serving as a basis for evaluating the performance of WRF and investigating potential SIP signatures.

### Comparing radar observations with radar observables

**Fig. 1:**
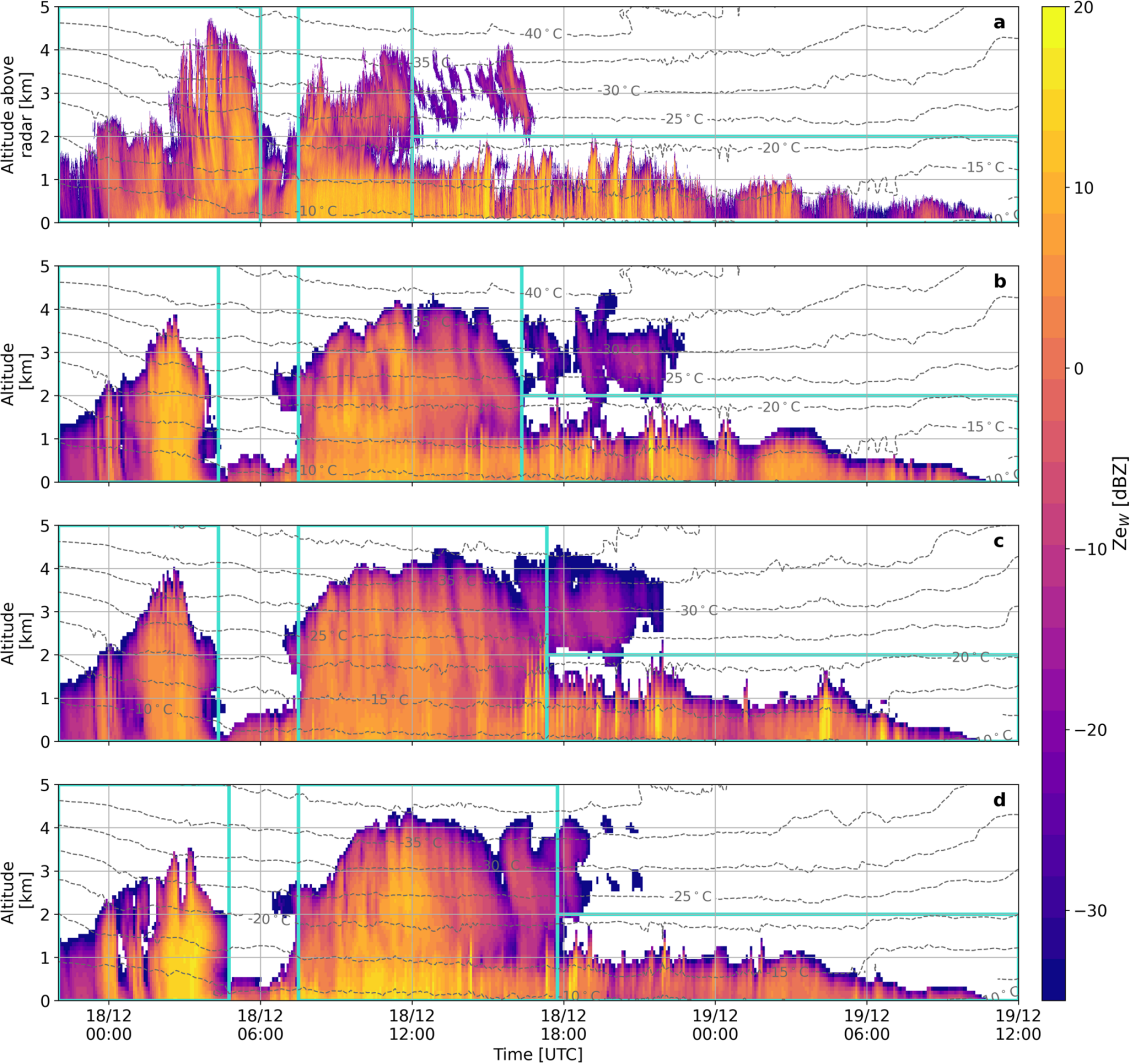
Comparison between observed and simulated reflectivities. Time-height plots of radar reflectivity *Ze*_w_ from December 17 (22:00 UTC) to December 19 (12:00 UTC), 2021, displaying (**a**) measurements by the WProf radar deployed at VL, and simulations coupling the CR-SIM radar simulator with the (**b**) CONTROL, (**c**) DEMOTT, and (**d**) ALLSIP simulations. Turquoise boxes indicate the three distinct cloud periods used to extract statistics. Grey contours in all panels represent temperature isotherms (in °C) from the CONTROL simulation in **a** and **b**, and the DEMOTT and ALLSIP simulations in **c** and **d**, respectively.

**Fig. 2:**
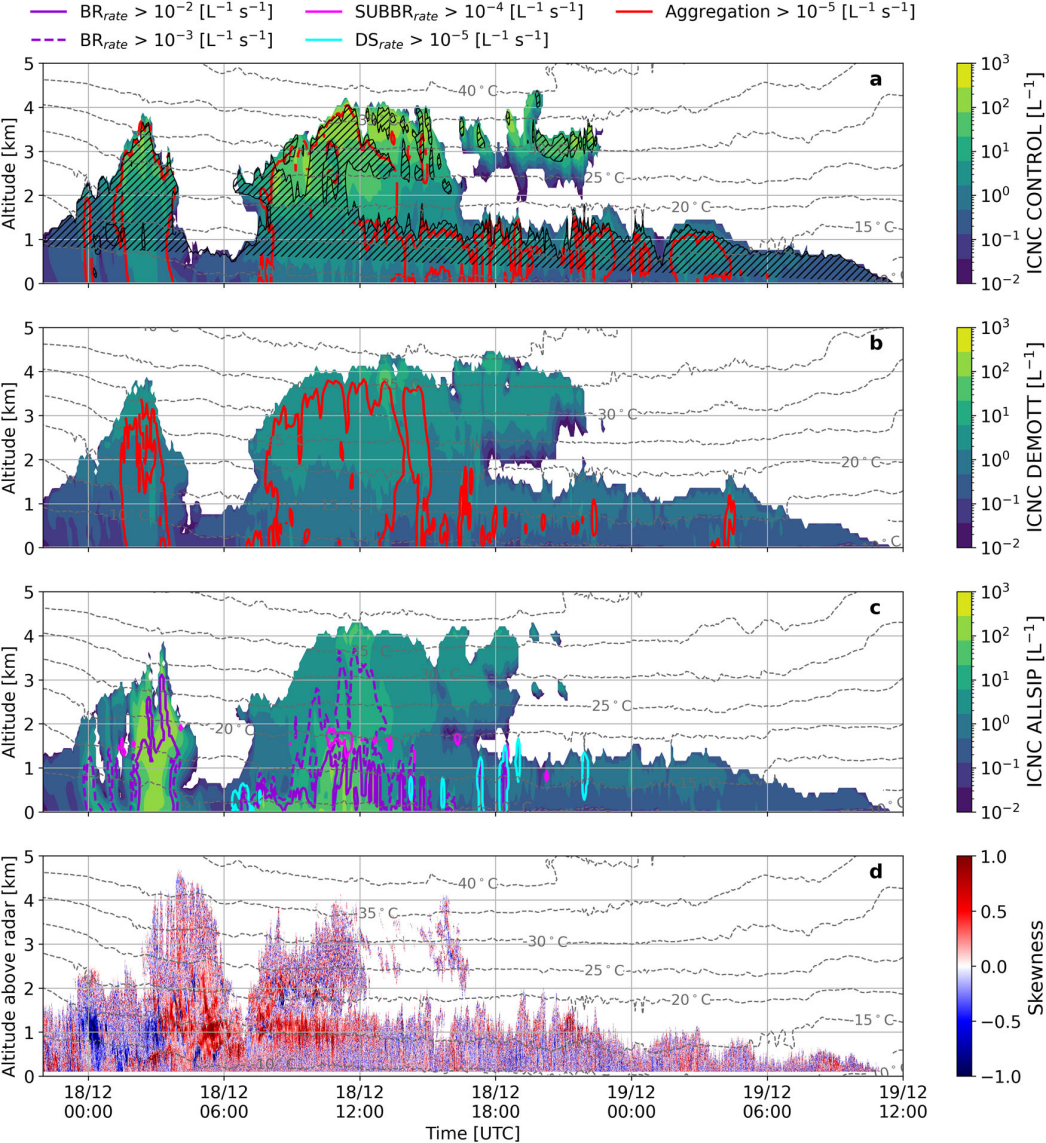
Ice crystal number distributions, secondary ice production rates and association with observed Doppler spectral skewness. Time-height plots of total ICNCs produced by the (**a**) CONTROL, (**b**) DEMOTT, and (**c**) ALLSIP simulations for the period December 17 (22:00 UTC) to December 19 (12:00 UTC), 2021. Grey contours in all panels represent temperature isotherms (in °C), while red contours in **a** and **b** show areas where snowflake aggregation rates exceed 10^−5 ^L^−1^ s^−1^. Note that snowflake aggregation tendencies are presented in absolute values, with the predictions from the ALLSIP simulation illustrated in Supplementary Fig. 5. Black hatched lines in **a** indicate regions that are supersaturated with respect to ice in the CONTROL simulation. In panel **c**, colored contours represent each active SIP rate: purple solid (dashed) contours indicate regions where BR rates exceed 10^−2^ (10^−3^) L^−1^ s^−1^, while cyan (magenta) contours show regions where DS (SUBBR) rates exceed 10^−5^ (10^−4^) L^−1^ s^−1^. The Doppler spectral skewness from WProf is also superimposed in panel **d**.

The vertical profile of the measured radar equivalent reflectivity factor (*Ze*_w_), which primarily reflects variations in hydrometeor size and total concentration, is presented in Fig. 1a. Note that all altitudes will be expressed as above ground level unless stated otherwise. WProf was deployed at the “Vathia Lakka” (VL) station, located on the lee-side of the mountain-top station, Helmos Hellenic Atmospheric Aerosol and Climate Change (HAC)^2^ ^58^, at an elevation of approximately 1850 m above mean sea level (AMSL). A low-pressure system associated with the passage of storm Carmel reached the CALISHTO measurement sites on the evening of December 17, 2021. The radar timeseries reveals three distinct cloud periods indicated by the turquoise boxes shown in Fig. 1a. The distinction between these three cloud periods in both measurements and simulations is based on the presence of seeding ice particles falling either from higher levels within the same cloud (internal seeding) or from an overlying cloud (external seeder-feeder), as summarized in Supplementary Fig. 1. The first cloud system exhibits a characteristic nimbostratus cloud structure, while the second one appears in a distinctive seeder-feeder cloud configuration, which is frequently observed in orographic environments^59^. This is further corroborated by the WRF simulations, as discussed in relation to Fig. 2a. Upon advection of the seeder cloud, a low-level orographic cloud persisted for almost an entire day.

To evaluate the simulations and understand the cloud microphysical processes occurring in the sampled radar volume, we configured the Cloud Resolving Model Radar Simulator (CR-SIM)^53^ to replicate the characteristics of WProf and coupled it with the outputs from the WRF grid cell nearest to the VL station. Three sensitivity experiments were performed with WRF (see Methods): CONTROL and DEMOTT account only for primary ice production (PIP). The former follows the temperature-dependent descriptions included in the default version of WRF, while the latter was updated with the more advanced aerosol-aware scheme developed by Demott et al. ^60^ and constrained by in-situ observations. ALLSIP simulation employs the aerosol-dependent scheme used in DEMOTT and further considers the action of four SIP processes: Hallett-Mossop (HM), ice-ice collisional break-up (BR), droplet-shattering upon freezing (DS) and sublimational break-up (SUBBR). HM is the sole SIP mechanism in the default M09 scheme following Reisner^61^. The description of BR and DS was based on the physically-based formulations developed by Phillips et al.^35^ and Phillips et al.^62^, respectively, while the effect of SUBBR follows the parameterization of Deshmukh et al.^43^. More details about the employed SIP parameterizations are provided in the Methods section.

It is worth mentioning that the timing of the first two simulated cloud events does not perfectly align with the remote sensing observations (Fig. 1). Coupling CR-SIM with the grid cells surrounding VL, or substituting the ERA5 (i.e., the fifth generation of the European Centre for Medium-Range Weather Forecasts atmospheric reanalysis) dataset with the National Centers for Environmental Prediction (NCEP) dataset did not significantly change the simulations (not shown). This discrepancy is likely attributed to errors in predicted wind fields and relative humidity with respect to ice (RH_i_; Supplementary Fig. 2b). Further evaluation of the model at higher altitudes is impeded by the intense snowfall during storm Carmel, which prevented the derivation of the wind profile from the wind lidar deployed at VL. Despite these model-observation discrepancies, WRF coupled with CR-SIM can capture the presence of the three consecutive cloud systems reasonably well, which is noteworthy given the complex microphysics and flow over the complex orographic terrain (Supplementary Fig. 3b).

The comparison between radar measurements and WRF simulations focuses solely on *Ze*_w_ (Fig. 1a–d), since the other radar observables were less accurately simulated by CR-SIM (not shown). Nevertheless, changes in the hydrometeor size distributions for example due to SIP^63^ will be directly mirrored in this radar product, which is essential for the purpose of our study. Replacing the default PIP scheme of WRF used in CONTROL (Fig. 1b) with the aerosol-aware scheme in DEMOTT (Fig. 1c), leads to a notable reduction in predicted *Ze*_w_ values especially at temperatures below −20 °C. This highlights the sensitivity of *Ze*_w_ profiles to changes in the PIP scheme. Activation of SIP mechanisms induces a distinct shift in simulated *Ze*_w_ towards higher values, evident at all altitudes during the nimbostratus cloud and more pronounced within the DGL temperature zone between −20 °C and −10 °C for the seeder-feeder cloud (Fig. 1d). A more detailed statistical summary of *Ze*_w_ for each cloud period is provided in Figs. 4b, 5b and Supplementary Fig. 4, that will help us determine which WRF configuration aligns most closely with the WProf measurements. Before delving into this comparison, it is essential to gain a better understanding of the microphysical processes shaping the simulated ice- and liquid-phase partitioning, and subsequently, the *Ze*_w_ values in the three WRF sensitivity simulations.

**Fig. 3:**
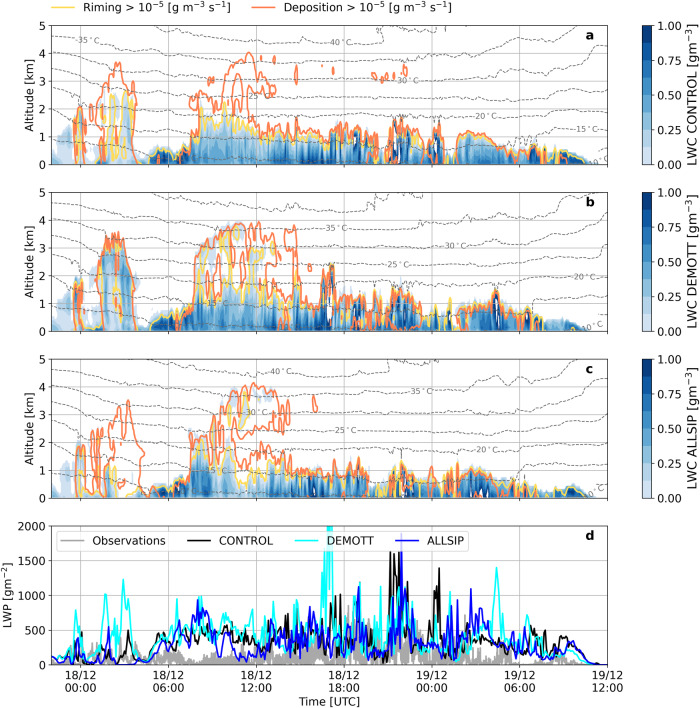
Liquid water content distributions from simulations and their evaluation against radiometer retrievals. Time-height plots of total LWC produced by the (**a**) CONTROL, (**b**) DEMOTT, and (**c**) ALLSIP simulations for the period December 17 (22:00 UTC) to December 19 (12:00 UTC), 2021. Grey contour lines represent temperature isotherms (in °C), while yellow (orange) contours delineate areas where riming (vapor deposition) rates exceed 10^−5 ^g m^−3^ s^−1^ across all panels. Panel **d** displays the time series of LWP retrieved at VL from the joint radiometer (grey solid line) and simulated by CONTROL (black line), DEMOTT (cyan line), and ALLSIP (blue line), respectively. Note that the simulated LWP takes into account both cloud droplets and raindrops.

The temporal evolution of vertical profiles for the total ICNC (cloud ice + snow + graupel) and liquid water content (LWC; cloud droplets + raindrops), as predicted by the three sensitivity simulations of WRF are illustrated in Fig. 2a–c and 3a–c, respectively. These profiles are extracted from the WRF grid point nearest to the VL station (i.e., the same location used for running the CR-SIM simulator). The hatched region in Fig. 2a delineates where water vapor is supersaturated with respect to ice, verifying the presence of two different ice seeding cloud configurations. During the first cloud period, frozen hydrometeors precipitate from the higher-level parts of the cloud, without experiencing ice subsaturation until below ∼1 km. In contrast, during the external seeder-feeder period, subsaturated air separates the orographic cloud from the synoptic cloud above. Note that, the supersaturated regions (with respect to ice) were not significantly affected when a different ice nucleation scheme was adopted in ALLSIP (Supplementary Fig. 5).

Within the first two ice seeding cloud periods, falling ice particles undergo effective mass gain, initially through vapor deposition, in the ice supersaturated cloud regions (Fig. 3a–c) at temperatures below −20 °C. As a result, these particles vary in size and experience different terminal velocities, enhancing their collision efficiencies and facilitating further growth through aggregation (Fig. 2a, b, Supplementary Fig. 5). Ice crystal growth through riming is also prevalent in the lower atmospheric levels of CONTROL (Fig. 3a) and extends to even colder temperatures in DEMOTT and ALLSIP during the seeder-feeder cloud period when higher-level clouds are not entirely glaciated (Fig. 3b, c). The role of INP description between simulations also carries important implications for the LWC and ICNCs at cold temperatures, which is further elaborated in Supplementary Text 1.

### SIP indications in Doppler spectra guided by WRF simulations

The absence of polarimetric radar observations during the CALISHTO campaign limits our ability to identify the ice hydrometeor shape and habits (i.e., columnar or plate-like crystals), which have proven valuable for the potential presence of SIP in previous radar-based studies^25,47^. Yet, in the following we demonstrate that by integrating information from the high-resolution modeling framework and ground-based radar observations, we can consistently attribute distinct radar signals to SIP or other microphysical processes without polarimetric data.

The ALLSIP sensitivity simulation of WRF accounting for the effect of ice multiplication, will help us determine the conditions favorable for SIP and the microphysical processes driving the radar observations. Figure 2c shows how activation of SIP modifies the cloud microstructure, shifting the yellowish shades (indicative of higher ICNCs) that were observed predominantly at temperatures below −20 °C in CONTROL, towards the warmer subzero temperature range. This leads to a reduction in the vertical availability of LWC (Fig. 3c). Although the WRF simulations tend to overestimate the radiometer-derived liquid water path (LWP) measured at VL, they capture the timing of the peaks in the timeseries, with ALLSIP more effectively reducing the simulated LWP (Fig. 3d). In the nimbostratus cloud period, ALLSIP predicts a mean LWP of 77 gm^−2^, approximately 40% (80%) lower than CONTROL (DEMOTT), bringing it closer to the observed mean value of 70 gm^−2^. However, during the seeder-feeder period, despite a 10% (35%) reduction in LWP in ALLSIP compared to CONTROL (DEMOTT), it remains insufficient to match the low observed mean LWP values of 45 gm^−2^ at VL.

The colored contours in Fig. 2c define regions where significant ice production occurs from SIP processes; BR dominates during the first two ice seeding events, with limited and localized contributions from DS mostly during the third cloud period (Fig. 2c). Raindrop sizes in the M09 scheme rarely surpass the 50 μm threshold needed for DS activation (Supplementary Fig. 6b), justifying the occurrence of spikes in the contours of DS production rates presented in Fig. 2c. SUBBR also shows highly localized effects when precipitating ice particles fall through subsaturated air layers (Supplementary Fig. 5). HM remains inactive during the simulation period, partially due to the colder temperatures at which the simulated clouds are formed, and also because of the imposed ice and liquid thresholds are not met (see Methods).

The prevalence of BR over SUBBR and DS is evident in its substantial ice production rates, surpassing 10^−3^ particles L^−1^ s^−1^ in both cloud periods. Its efficiency exhibits a tenfold increase in production rates when the nimbostratus cloud top rises below the −25 °C isotherm or in the feeder region of the seeder-feeder cloud, particularly at temperatures exceeding −15 °C (Fig. 2c). Another noteworthy observation is that the snowflake aggregation contours (Supplementary Fig. 5) consistently envelop the BR contours (Fig. 2c), suggesting that inside the DGL, collisions of aggregated dendrites can trigger SIP through BR. Although the ice habit is not explicitly resolved in the M09 microphysics scheme of WRF, the number of fragments described by the Phillips et al.^64^ parameterization – employed to represent the BR process – shows a triangular relationship with temperature, peaking at around −15 °C, which justifies the peak in its efficiency inside the DGL.

In the single-layer orographic cloud (3rd cloud period), where aggregation is not favored in ALLSIP (Supplementary Fig. 5), BR is completely inefficient and WRF fails to adequately capture the observed spikes of enhanced *Ze*_w_ (Fig. 1a, d, Supplementary Fig. 4). CONTROL seems to better capture the observed *Ze*_w_ spikes (Fig. 1b), which implies uncertainties related to the representation of PIP during this period. Indeed, important INP types (e.g., biological INPs), are not represented by the DeMott^60^ scheme. Modeling uncertainties during this period might also be attributed either to the uncertain representation of SIP mechanisms (e.g., DS) or to the neglected effect of blowing snow^65^ or pre-activation of INPs^17,66,67^. A more in-depth discussion is provided in Supplementary Text 2.

**Fig. 4:**
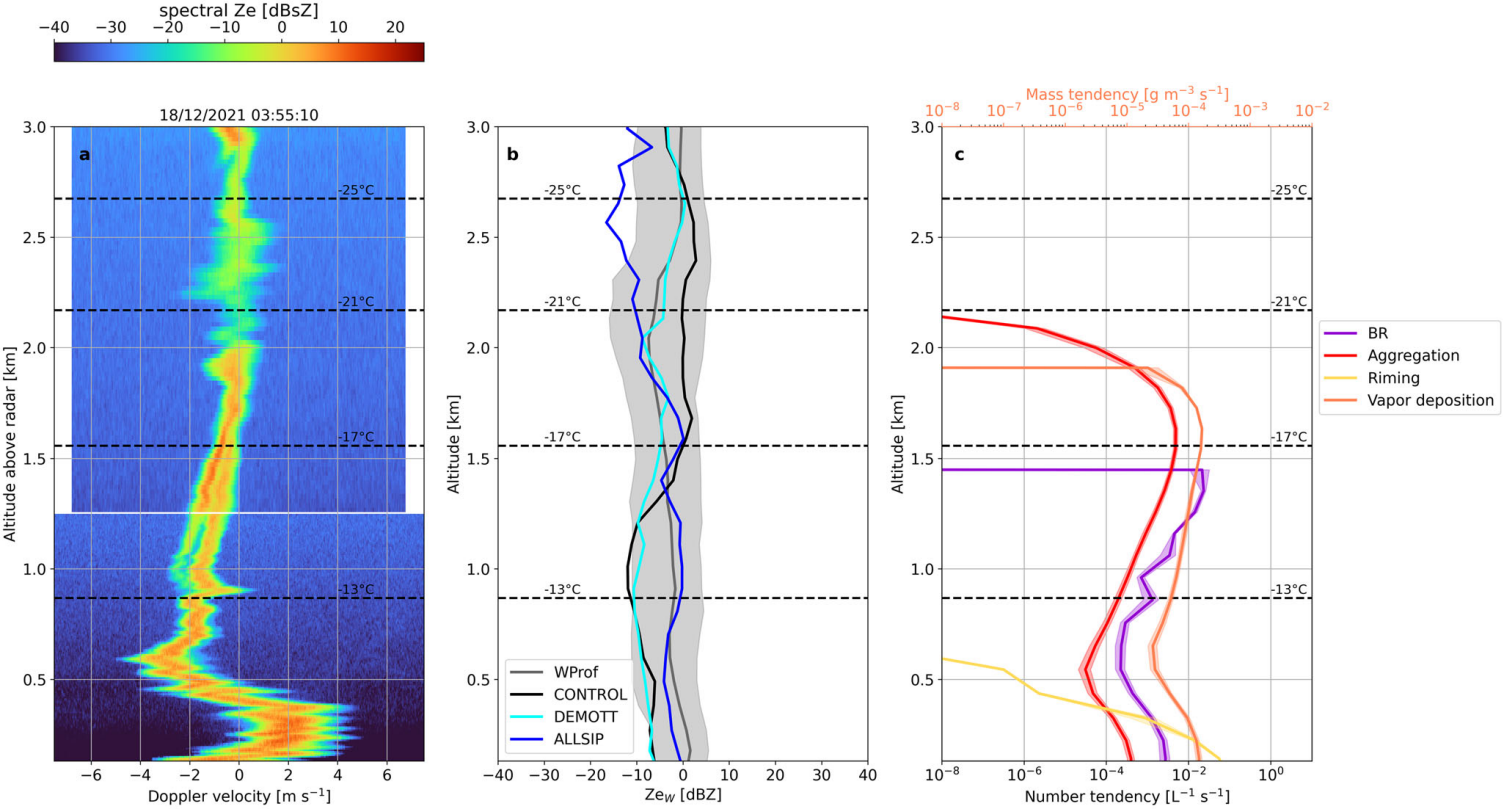
Radar observations versus simulations for the nimbostratus cloud period. Synergistic insights from WProf radar Doppler spectra and WRF predictions: (**a**) Example of the WProf reflectivity spectrogram obtained on December 18 at 03:55:10 UTC, during the nimbostratus cloud period. A horizontal white line around 1.2 km marks a sampling gap in a small radar volume between the first and second chirp. Note that 1dBsZ = 10log_10_ (1 mm^6^ m^−3^ (ms^−1^)^−1^); **b** Median vertical profiles of observed and simulated radar reflectivity extracted during the nimbostratus cloud period. The grey line represents median WProf observations, while the black, cyan, and blue lines denote the results from the CONTROL, DEMOTT, and ALLSIP simulations, respectively; **c** Median vertical profiles (extracted from the ALLSIP simulation over a 10-min time window centered around the chosen spectrogram) of the number tendency due to BR (purple line) and snow aggregation (red line) displayed on the lower horizontal axis, while the mass tendencies due to riming (yellow) and vapor deposition (orange) are shown on the upper horizontal axis. Shaded regions correspond to the IQR, while temperature contours overlaid in these panels are from the ALLSIP simulation. Note that the tendency due to snow aggregation in panel **c** is presented in absolute values.

Despite these challenges, it becomes evident that both ice seeding periods create SIP favorable conditions, implying that the ALLSIP simulation can be used to investigate whether characteristic radar signatures can be linked to the presence of specific SIP mechanisms. We will therefore focus on two specific moments of the timeseries – one from the nimbostratus and the other from the seeder-feeder cloud periods trying to identify potential SIP fingerprints within observed radar Doppler spectra. Figure 4a presents a Doppler spectrogram measured on December 18 at 03:55:10 UTC during the nimbostratus cloud period. This spectrogram is derived from a period with persistent bimodalities in the WProf observations, as illustrated in the spectra timeseries provided for a specific altitude in Supplementary Fig. 7. The chosen spectrogram highlights a turbulent region between 2 and 2.5 km altitude, below which a clear bimodal distribution appears at around 1.6 km, signifying two hydrometeor populations (Fig. 4a).

Median statistics for this cloud period are summarized in Fig. 4b, with gray shaded regions indicating the observed interquartile range (IQR). CONTROL overestimates *Ze*_w_ from around −25 °C to −16 °C, while underestimates *Ze*_w_ at warmer subzero temperatures. The updated PIP scheme in DEMOTT agrees better with observations at temperatures colder than ∼−17 °C, yet it still fails to achieve the higher *Ze*_w_ values observed at warmer temperatures. In contrast, ALLSIP enhances the simulated *Ze*_w_ by over 10 dBZ at these temperatures, reducing the discrepancy with WProf measurements. Analysis of simulated ice particle size distributions (Supplementary Fig. 8) reveals that, closer to the ground (∼700 m above ground level), ALLSIP predicts more than two (one) orders of magnitude elevated ICNCs compared to CONTROL (DEMOTT). In terms of large particles dominating the radar reflectivity, ALLSIP predicts tenfold higher snow particle concentrations compared to the other two sensitivity simulations (Supplementary Fig. 8e), likely from increased cloud-ice-to-snow autoconversion. However, at colder temperatures, particularly below −20 °C, *Ze*_w_ values simulated by ALLSIP fall below the observed IQR. Even though SIP is expected to increase *Ze*_w_ as a result of the elevated ICNCs, yet a more important factor determining the radar reflectivity is the size of the hydrometeors. In higher atmospheric levels where SIP is initiated, a shift towards smaller particle sizes could therefore explain why ALLSIP predicts lower *Ze*_w_ compared to CONTROL and DEMOTT.

Figure 4c illustrates median tendency profiles for several microphysical processes simulated by ALLSIP within a 10-min timeframe of the observed WProf spectrogram (Fig. 4a). Ice particles grow through vapor deposition and aggregation while falling through the atmosphere. The primary mode detected by the cloud radar could therefore be attributed to dendritic and/or aggregated ice particles. Furthermore, the mean Doppler velocity (MDV) of the primary peak reaches up to ∼−2.5 m s^−1^ at 1 km altitude (Fig. 4a), which is significantly higher than the typical terminal velocities of aggregates at around 1 ms^−1^ ^68^. Although large MDV could be caused by heavily-rimed ice structures, WRF simulations do not suggest the presence of supercooled liquid water (Fig. 3c). The high MDV is likely from the influence of downdrafts, and the effects of prevailing high horizontal winds (Supplementary Fig. 2c) and potential deviations in the vertical radar setup alignment^69^.

At temperatures above −17 °C a secondary mode emerges in the radar Doppler spectrogram with a reflectivity of −0.4 dBZ (Fig. 4a). The pronounced reflectivity of the peak, alongside its broad spectral range, rule out the possibility of its origin being supercooled liquid droplets, as such droplets typically exhibit lower values below −15 dBZ^46^. Here it is worth noting that a single Doppler spectrogram at a specific timestep does not necessarily reflect the microphysical trajectory of a particle population^46^. The fast-falling spectral mode in Fig. 4a may, indeed, result from overlapping particle trajectories as they are advected toward the measurement site. Based on the ALLSIP predictions, the emergence of the secondary mode in the Doppler spectrogram almost coincides with enhanced aggregation and BR in the model, the latter peaking above −17 °C (Fig. 4c). Note that, flight measurements within nimbostratus clouds over China revealed the presence of fragmented dendritic ice crystals, implying the dominant role of BR particularly between −10 °C and −15 °C^70^. Efficient growth of sedimenting ice particles inside the DGL promotes differential settling, increasing the likelihood of collisions, which in turn drives both aggregation and BR. These two processes exhibit consistent alignment across all altitudes in the atmosphere. While snowflake aggregation can be an efficient ICNC sink in MPCs, Fig. 4c reveals that aggregation drives SIP through BR, which in turn compensates for the depletion of ice crystals and may even enhance them. Below ∼600 m, BR-induced ice particles can grow efficiently through riming (Fig. 4c) and possibly WBF, depleting cloud liquid water in lower atmospheric levels, improving agreement with radiometer-derived LWP compared to CONTROL and DEMOTT (Fig. 3d). The distinct spectral modes broaden and converge below ∼1 km, indicating an “advection-type” effect (although this could also relate to atmospheric turbulence or the imperfect vertical beam alignment during this instance).

**Fig. 5:**
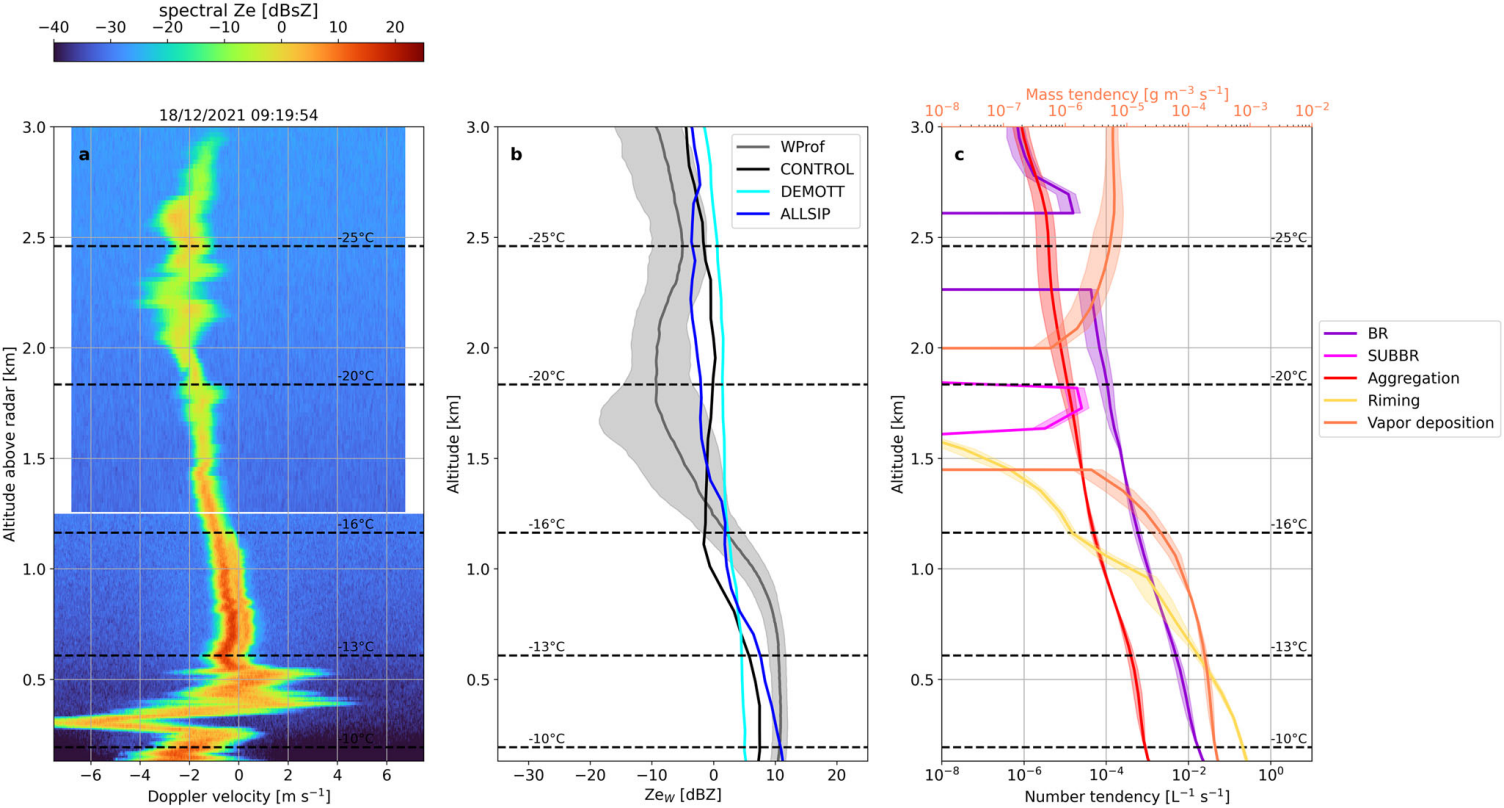
Radar observations versus simulations for the seeder-feeder cloud period. Panels **a**–**c** display data similar to those in Fig. 3, but from the seeder-feeder cloud event on December 18 at 09:19:54 UTC. The magenta line shown in panel **c** indicates the median vertical profile of number tendency due to SUBBR displayed on the lower horizontal axis.

Moving to the seeder-feeder cloud period, in the selected WProf spectrogram (Fig. 5a) we can again discern the primary hydrometeor population, which, as indicated by the WRF simulations, gains mass through vapor deposition and aggregation while falling from the seeder cloud (Fig. 5c). A clear secondary mode becomes evident in the feeder cloud at temperatures above −16 °C (Fig. 5a) – a region where ALLSIP predicts the presence of BR-generated particles (Fig. 5c). A high reflectivity of −1.0 dBZ together with its quite wide spectral signature indicate that the slow-falling spectral subpeak corresponds to cloud ice particles rather than supercooled liquid droplets.

The median profiles extracted from this cloud period reveal two distinct *Ze*_w_ profile characteristics (Fig. 5b). In the feeder part of the cloud, the measured *Ze*_w_ saturates likely from non-Rayleigh scattering^71^ by large ice particles with sizes comparable to the WProf wavelength (3.2 mm). Indeed, the simulated size distribution of snow particles, supports the presence of large particles exceeding 1 mm at altitudes below 1 km (Supplementary Fig. 6a). At these altitudes BR aligns with aggregation, but consistently generates an order of magnitude more particles L^−1^ s^−1^ than aggregation depletes from snowflake number loss (Fig. 5c). The efficiency of BR maximizes closer to the surface, yielding almost 2 × 10^−2^ small ice fragments L^−1^ s^−1^. Even though CONTROL and ALLSIP produce comparable snow number concentrations, the latter yields almost 2 orders of magnitude elevated cloud ice particle concentrations (Supplementary Fig. 9d). The subsequent growth of these particles via vapor deposition and riming (Fig. 5c) boosts the simulated *Ze*_w_ values leading to better agreement not only with the WProf reflectivity profile (Fig. 5b) but also with the LWP measurements during this period (Fig. 3d).

At higher altitudes and temperatures between approximately −16 °C and −25 °C, WProf measured reduced *Ze*_w_ that is not reproduced by any WRF simulation (Fig. 5b). This discrepancy is likely from the presence of a dry layer separating the two clouds (Fig. 2a and Supplementary Fig. 5) that does not backscatter much signal to the radar, or in which ice particles are decreasing in size (and hence in reflectivity) because of sublimation, especially under subsaturated conditions. The timing and microphysics inside this cloud free region is likely more challenging to be captured by all model set-ups examined, but this does not appear to have a significant impact on ground precipitation and SIP. CONTROL generates cloud ice (Supplementary Fig. 9a) and snow particle concentrations (Supplementary Fig. 9b) one order of magnitude higher than DEMOTT at an altitude of ∼2.2 km, which is nearly one (two) orders of magnitude elevated cloud ice (snow) concentrations compared to ALLSIP. The elevated concentrations of larger ice particles are probably causing the overestimated *Ze*_w_ values in CONTROL and DEMOTT. At these temperatures, initiation of BR in the ALLSIP simulation is found to shift the particle distributions towards smaller sizes, effectively moving the simulated *Ze*_w_ values closer to the observed IQR at the appropriate altitude and timing. Additionally, it is worth noting the contribution of SUBBR within the subsaturated air layer that separates the seeder from the feeder cloud regions, generating up to 10^−5^ particles L^−1^ s^−1^. This aligns with findings presented in Deshmukh et al.^43^ (see their Fig. 14).

### Insights from spectral skewness inside the DGL

Examining a limited number of Doppler spectrograms, while informative, poses challenges in inferring long-term statistics for SIP occurrences. Scrutinizing the timeseries of spectral skewness (the fourth moment of the radar Doppler spectrum), reveals regions with either positive or negative skewness during the first two cloud periods (Fig. 2d). Changes in the sign of skewness imply shifts in the balance of different hydrometeor populations within the sampled radar volume. Negative skewness, indicative of spectra skewed towards more massive, faster-falling particles (with the sign convention employed here), is observed at temperatures higher than −15 °C before 03:00 UTC on December 18. Following the ALLSIP predictions, this can be attributed to significant ice particle growth through aggregation (Supplementary Fig. 5) and riming (Fig. 3c).

Sizable regions with positive spectral skewness are identified during both ice seeding cases between the −10 °C to −20 °C isotherms within the DGL (Fig. 2d). During the first cloud period, persistent red shading appears when the nimbostratus cloud gains vertical extent (after 03:00 UTC). This feature seems to coincide with the emergence of the secondary, slower-falling mode shown in the bimodal spectrogram of Fig. 4a, confirming that the picked bimodal spectrogram is not an isolated feature (see also Supplementary Fig. 7). Moreover, the observed increase in spectral skewness aligns both spatially and temporally with periods when ALLSIP predicts enhanced BR (Fig. 2c) and aggregation (Supplementary Fig. 5). This co-occurrence implies that, at least in the studied snowfall event, positively skewed radar Doppler spectra between −10 °C and −20 °C can serve as a fingerprint for SIP via collisions between delicate dendritic and/or aggregated ice structures within the DGL. This is further corroborated by the consistent ALLSIP predictions of *Ze*_*w*_ during this period (Fig. 4b).

Similar patterns apply to the external seeder-feeder cloud period, showing an extensive region with positive skewness, mainly between the −15 °C and −20 °C isotherms (Fig. 2d). This red shading in the skewness timeseries aligns with activation of BR in the ALLSIP simulation (Fig. 2c) and the emergence of bimodal spectrograms in the radar observations (e.g., Fig. 5a). This reaffirms our prior hypothesis regarding the connection between positive skewness, spectral bimodality (Fig. 5a), and microphysical processes such as BR and aggregation (Fig. 3c and Supplementary Fig. 5) within the DGL.

## Discussion

The effect of BR-driven SIP in orographic clouds during ice seeding events is now well-established^63,72^. In the simulated snowfall event, BR initiates near the upper edge of the DGL temperature regime (−20 °C to −10 °C), amplifying cloud ice and snow concentrations by 1-2 orders of magnitude. This contributes to increased snowfall near the ground, resulting in elevated radar reflectivities, replicable solely through the WRF simulation considering SIP.

The presence of multi-modalities in the radar Doppler spectra has long offered the possibility of improved understanding of the intricate microphysical processes taking place in MPCs. Spectral bimodalities within the DGL have already been associated with new ice formation^45,48,73^. The SIP-aware WRF simulations demonstrated that these spectral signatures coincide with the initiation of aggregation and BR in the model, the simultaneous enhancement of which has already been highlighted in modeling^63,74^ and remote sensing studies^45^. The faster-falling spectral mode was associated with the aggregate population, while distinct secondary modes emerged at temperatures higher than −17 °C, coinciding with enhanced BR and aggregation in WRF and showing good temporal and spatial alignment with an increase in the observed skewness timeseries. Persistent positive skewness arises within the DGL due to the shift of spectra toward the slower-falling peak after SIP initiation.

Our findings propose strong connections between positive skewness, Doppler spectral bimodalities, aggregation, and BR within the DGL in the SIP favorable temperature regime. In a winter frontal case study, von Terzi^45^ unveiled spectral asymmetry associated with the rapid increase in Doppler spectral skewness at temperatures above −18 °C, attributed to new small ice particle formation likely due to SIP processes within the DGL. At warmer subzero temperatures, Giangrande et al.^49^ also linked bimodal spectra skewing toward slower falling particles with the formation and growth of ice needles. Our analysis indicates that even without involving polarimetry, a first, qualitative inference of the BR-active cloud regions within the DGL can be achieved simply by focusing on the skewness timeseries. Expanding our analysis beyond the limited spatial and temporal scales considered, is critical to establishing the statistical significance of identified SIP signatures. For a quantitative investigation, synergy with SIP-aware models or developing remote-sensing techniques, as presented in Luke et al.^27^, applied to Arctic MPCs at temperatures above −10 °C, would be imperative. Ground-based radar observations are ubiquitous even in remote regions like the Arctic^27^ and Antarctica^75^, providing a unique tool for studying the vertical distribution of whole cloud volumes and their time evolution.

Our results reveal that, even without polarimetric radar observations, valuable information about SIP occurrence can be deduced by directly comparing observed full Doppler spectra and standard radar moments with outputs from a forward radar simulator coupled with high-resolution model simulations that consider SIP. The fusion of models with observations developed here offers a promising avenue for retrieving microphysical mechanisms from long-term remote-sensing records. This is expected to not only enhance our comprehensive global understanding of SIP but also help in pinpointing potential discrepancies in the representation of SIP physics in models.

## Methods

### CALISHTO campaign

The CALISHTO field campaign (https://calishto.panacea-ri.gr/) took place between October 2021 and March 2022 at Mount Helmos, Greece, with the primary objective of enhancing our understanding of the processes involved in orographic MPC formation and evolution^76–78^. During CALISHTO, extensive in-situ and remote sensing observations were carried out at three different altitudes and locations. Meteorological, aerosol, and cloud measurements were taken at the mountain-top station, (HAC)^2^, located at 2314 m AMSL (37°N 59’ 2.4”, 22°E 11’ 45.6”) (Supplementary Fig. 3)^58^. At the VL station, located on the lee side of (HAC)^2^, aerosol and cloud measurements were conducted using remote sensing techniques. At the lower altitude site, ∼1700 m AMSL, a multi-wavelength depolarization lidar was used to sample vertical profiles of aerosol and cloud properties.

Cloud in-situ measurements were conducted at (HAC)^2^ using the Gerber Particulate Volume Monitor (PVM-100, Gerber Scientific Instruments Inc.)^79^. This instrument is designed to measure the LWC, particle surface area (PSA), and derive the droplet effective radius (*r*_eff_) for ambient clouds. To obtain these measurements, a diode-emitted laser beam is directed along a 40-cm path, and the scattered light in the open air along the path is converted into a signal after passing two spatial filters. The first filter converts scattered light to a signal proportional to the particle volume density (or LWC), while the second filter produces a signal proportional to the PSA. By analyzing the ratio of these two quantities, *r*_eff_ can be derived for droplet diameter from 3 to 45 µm. The uncertainty of LWC is 10% for this diameter range. The PVM-100 instrument has undergone testing and inter-comparison with other instruments during ACTRIS (Aerosol Cloud and Trace gases Research Infrastructure) activities^80^. For the purposes of this study, LWC and *r*_eff_ data collected by the PVM-100 were utilized to derive the cloud droplet number concentration (*N*_d_) based on the formulation presented in Brazda et al.^81^ (see their Equation 4).

Aerosol size distributions over the size range between 0.25 and 32 μm were measured at (HAC)^2^ by an Optical Particle Counter (OPC; GRIMM Technologies Inc., Model 1.109), which provides real-time aerosol characterization including 32 channels. In this study, OPC diameter (*d*_opc_) was converted into aerodynamic diameter (*d*_aer_) using the formula: ${d}_{{\rm{aer}}}={d}_{{\rm{opc}}}{\left(\frac{\rho }{\chi }\right)}^{0.5}$, assuming a shape factor of *χ* = 1.1 and a particle density of *ρ* = 2.0 g cm^−3^ ^82,83^.

### Remote sensing observations

At the VL station, a frequency-modulated continuous wave (FMCW) W-band Doppler spectral zenith profiler (WProf)^57^ was deployed. Operating at a frequency of 94 GHz, WProf allows for measurements up to approximately 10 km above ground level. Vertically, WProf employs three chirps, each with a respective range resolution of 7.5 m, 16 m, and 32 m. WProf settings are summarized in Supplementary Table 1. For this study, we utilized full Doppler reflectivity spectra and corresponding moments. These moments include the *Ze*_w_, MDV and skewness. An attenuation correction has been applied to the W-band radar reflectivities, to facilitate their comparison against the forward simulation products. To do so, the radiative transfer model PAMTRA^84^ was used to simulate both gaseous and cloud liquid water attenuation at 94 GHz. The vertical profiles of the necessary atmospheric and liquid water profiles were obtained from the WRF model set-up, which includes the most advanced description of both primary and secondary sources of ice crystals (see ALLSIP simulation described below).

In addition to the radar variables, WProf offers the capability to estimate the cloud LWP using a retrieval algorithm presented by Billault-Roux and Berne^85^. This algorithm uses the brightness temperature measured by a joint 89-GHz radiometer, in combination with available meteorological data such as temperature, pressure, and reanalysis data as well as geographical information (i.e., latitude, longitude). The relative error in the retrieved LWP values was determined to be 18% for cloudy cases (i.e., LWP > 30 g m^−2^).

### WRF set-up

We utilized WRF version 4.0.1, incorporating augmented cloud microphysics to account for additional SIP mechanisms^30,63,86^, to model the current case study. Our model configuration consisted of three two-way nested domains (Supplementary Fig. 3a) with horizontal resolutions of 12 km, 3 km, and 1 km, respectively. The parent domain encompassed a 148 × 148 grid centered over the (HAC)^2^ station. The second and third domains consisted of 241 × 241 and 304 × 304 grids, respectively. All domains employed the Lambert conformal projection, suitable for mid-latitudes. We implemented a refined vertical grid spacing, following the approach proposed by Vignon^54^, employing 97 vertical eta levels up to a model top of 50 hPa (∼20 km). Note that the employed model setup was consistent with the one utilized for wintertime orographic clouds in the Swiss Alps^63^.

The WRF simulations started on December 17, 2021, at 00:00 UTC, providing 22 h of spin-up time before the passage of storm Carmel over the region of interest. This low-pressure system was associated with polar airmasses originating from northern Europe (Supplementary Fig. 10), bringing a significant temperature decrease, stormy winds, and heavy snowfall to most parts of Central and Southern Greece. The temperature drop and the prevailing strong-wind conditions are illustrated in Supplementary Fig. 2, where they are compared against surface meteorological variables obtained from the weather station at (HAC)^2^ to evaluate the performance of the model. Our analysis covers the period until December 19, 2021, at 12:00 UTC. We employed a time step of 36 s in the parent domain, which decreased to 9 s in the second domain and 3 s in the third domain. The output frequency was set at every 5 min. Information about the physics options employed here are provided in Supplementary Text 3.

### Microphysics scheme and sensitivity simulations

Cloud microphysics is parameterized using the M09^56^ scheme of WRF. This scheme utilizes a double-moment approach to represent the characteristics of raindrops, cloud ice, snow, and graupel particles by predicting both their mass and number concentrations. However, for cloud droplets, a single-moment approach is employed, necessitating the specification of a constant *N*_d_. During the passage of storm Carmel, a power outage caused by severe weather conditions disrupted the PVM-100 measurements. In our study, we opted for an *N*_d_ value of 100 cm^-3^. This choice aligns with the temperature-dependent median *N*_d_ spectrum observed by PVM-100 throughout the CALISHTO campaign and is also consistent with observations at the high-altitude station of Jungfraujoch in the Swiss Alps^87^.

The M09 scheme incorporates different ice formation processes. Homogeneous freezing is considered for temperatures below −40 °C, while heterogeneous ice nucleation is initiated below −4 °C. The latter accounts for various temperature-dependent mechanisms, including immersion freezing of cloud droplets and raindrops^88^, contact freezing^89^, and condensation/deposition freezing nucleation^90^. The default PIP scheme of WRF was used to perform the CONTROL sensitivity simulation. However, when comparing the predicted INPs derived offline using the simplified temperature-dependent formulations of WRF with two-month INP measurements taken between −28 °C and −23 °C at (HAC)^2^, a significant overestimation of up to three orders of magnitude was observed (Supplementary Fig. 11b, c, Supplementary Text 4). As a more advanced alternative, DeMott^60^ (DM10) developed an aerosol-aware scheme that accounts for the concentration of aerosols with sizes larger than 0.5 μm aerodynamic diameter (*n*_aer,0.5_) and temperature. The DM10 parameterization yielded more realistic offline INP concentrations, with predictions that agree with observations within a factor of three (Supplementary Fig. 11a) for more than 70% observed data points. Therefore, in the DEMOTT sensitivity simulation, we replaced the default PIP scheme of WRF with the DM10 parameterization. In our case an *n*_aer,0.5_ of 0.30 scm^−3^ was prescribed, as dictated by the mean OPC measurements taken during the simulation period. Note that in DEMOTT, the Bigg^88^ parameterization was still kept active to account for the freezing of big raindrops.

The final sensitivity simulation analyzed in this study, referred to as ALLSIP, incorporates both PIP and SIP processes, with the former following the advanced DM10 parameterization. The M09 scheme, similar to other microphysics schemes in NWP models, includes the representation of only one SIP process: HM. This process is parameterized following Reisner et al.^61^, which accounts for the production of ice splinters within the HM temperature range. It occurs when supercooled droplets or raindrops collide and freeze onto snow or graupel particles. Provided that a certain threshold in the mixing ratios of the involved ice and liquid hydrometeors is exceeded, the efficiency of this process is regulated by a temperature-dependent scaling factor which allows for a maximum production rate of 350 splinters mg^−1^ of accreted liquid, at around −5 °C^61^. The splinter production rate decreases linearly towards the edges of the HM zone and becomes zero outside this range.

The BR mechanism is an additional SIP process considered in ALLSIP. In the M09 scheme, BR follows the parameterization developed by Phillips et al.^35^, which has been shown to provide realistic representation of ICNCs in orographic MPCs^63^. A detailed implementation of the BR mechanism in M09 is described elsewhere^30^. The number of ice fragments generated from collisions among the three ice hydrometeor species is determined by factors such as collisional kinetic energy, size, rimed fraction, and ice habit of the particles involved. While M09 does not explicitly resolve the rimed fraction and ice habit, assumptions are made to account for their influence. The impact of the prescribed rimed fraction has been previously investigated^30,91^, and a sensitivity experiment in the current case study revealed that a rimed fraction of 0.2 aligns better with observed cloud systems (not shown). Higher degrees of riming led to unrealistically high ICNCs, particularly within the lower cloud layers. Activation of BR in the model requires a nonzero mass of raindrop or cloud droplet to be rimed onto the ice particle, leading to fragmentation. It is important to note that the original BR scheme was designed for ice particles larger than 500 μm. According to Phillips et al.^35^, when dealing with smaller ice particle sizes, it is advisable to set them to the nearest limit within the specified range. Consequently, we limit the efficiency of BR to particles with a characteristic size exceeding 100 μm. Regarding the ice habit, the Phillips parameterization provides two formulations depending on the prevailing temperature range. Dendritic particles are considered between −17 °C and −12 °C, while non-dendritic planar ice particles are assumed outside this temperature range. Minimal sensitivity has been found with respect to the prescribed ice habit^91^, and we thus adopt planar ice particles, which capture a wider temperature range and are valid for a broader range of particle shapes. All secondary ice fragments resulting from the BR mechanism are classified as cloud ice.

Another SIP process accounted for in ALLSIP is the DS mechanism. A detailed description of how M09 scheme was updated to include this process is provided elsewhere^63^. DS involves two collision modes^62^. In the first mode, freezing and subsequent shattering occur when a supercooled raindrop collides with a less massive cloud ice particle or when an INP triggers freezing in immersion mode. The number of fragments generated in this mode are multiplied by the product of droplet freezing and shattering probabilities, being described by cubic interpolation functions^62^. The former is set to unity for temperatures below −6 °C and zero for temperatures above −3 °C, while the latter depends on the size of the raindrop, being 0 for sizes smaller than 50 μm, 1 for sizes larger than 60 μm. The second mode, involves collisions between raindrops and larger ice particles such as snow or graupel^92^. These collisions produce tiny ice fragments, which are introduced as cloud ice in the number conservation equations. Larger fragments are classified depending on the specific collision that triggered the freezing process of raindrops, which will in turn determine whether they will be treated as graupel, snow, or frozen drops.

The last SIP process considered in ALLSIP is SUBBR, which occurs when dendritic or heavily rimed particles sublimate under subsaturated conditions within downdrafts, resulting in the detachment of ice parts (e.g., branches from dendrites) from the parent ice particle^42^. A recent study^43^ introduced two empirical formulations for the SUBBR of graupel and dendritic snow. When implemented into the M09 scheme, the former parameterization is valid throughout all temperatures provided that the RH_i_ is less than 100%. The latter is enabled at temperatures between −20 °C and −10 °C, where the dendritic ice habit of snow particles is favored^93^. The number of fragments generated after SUBBR (${N}_{{\rm{SUBBR}}}$) is determined by the product: ${N}_{{\rm{SUBBR}}}=K{M}^{0.5702}$, where *K* is a function of the initial size of the particle, ambient RH_i_, and a ventilation factor associated with the fall speed of the particle, while *M* is the sublimated mass described by the M09 scheme. More details about this empirical parameterization can be found elsewhere^43^.

### The CR-SIM forward radar simulator

Forward simulators are valuable tools for converting model output into quantities that can be directly compared with observations from remote sensing instruments. This enables a more accurate assessment of the agreement between model predictions and real-world data. In our study, we utilized the outputs from the 3 WRF sensitivity simulations (i.e., CONTROL, DEMOTT, and ALLSIP) as input for the Cloud Resolving Model Radar Simulator (CR-SIM) version 3.32^53^. CR-SIM is compatible with various microphysics schemes of WRF and has previously been employed to evaluate the performance of polar WRF in representing Southern Ocean MPCs and snowfall microphysics^54,55^. The T-matrix method is used in CR-SIM to calculate the scattering properties of simulated frozen and liquid hydrometeors, which are then organized into look-up tables. In our study, CR-SIM was configured as a vertically profiling radar operating at 94 GHz, matching the frequency of the WProf deployed at VL. The radar beamwidth and range resolution were also adjusted to align with the characteristics of the actual instrument. The CR-SIM was run using a specific model grid point located closer to the VL station. The idealized simulated radar variables (i.e., after correction for the total hydrometeor attenuation) are then provided at each vertical model grid cell, facilitating straightforward comparisons with real observations.

### Supplementary information

Supplementary tables, texts and figures to the main article

## Data Availability

All simulation data presented in this study along with the in-situ and remote sensing observations are available at 10.5281/zenodo.10838606 (ref.^94^).

## Code availability

The original version of WRF used in this study (version 4.0.1) is open source and can be accessed at (https://github.com/wrf-model/WRF). The source code for CR-SIM is available at https://you.stonybrook.edu/radar/research/radar-simulators/. The code needed to generate the figures in this paper is provided at 10.5281/zenodo.10838606 (ref.^94^).
