# Supplementary material for: Unraveling ice multiplication in winter orographic clouds via in-situ observations, remote sensing and modeling
Source: NPJ Clim Atmos Sci. 2024 Jun 22;7(1):145. doi: 10.1038/s41612-024-00671-9 (PMC11193683; doi:10.1038/s41612-024-00671-9)
Supplement: Supplementary file 1 — Supplementary tables, texts and figures to the main article [file 41612_2024_671_MOESM1_ESM.pdf]

Supplementary material to

“Unraveling secondary ice production in winter orographic clouds through a synergy of in-situ observations, remote sensing and modeling”

Paraskevi Georgakaki[1], Anne-Claire Billault-Roux[2], Romanos Foskinis[3,4,5,1], Kunfeng Gao[1], Georgia Sotiropoulou[6,1], Maria Gini[5], Satoshi Takahama[1], Konstantinos Eleftheriadis[5], Alexandros Papayannis[1,3], Alexis Berne[2], and Athanasios Nenes[1,4*]

[1]Laboratory of Atmospheric Processes and their Impacts (LAPI), School of Architecture, Civil & Environmental Engineering, Ecole Polytechnique Fédérale de Lausanne, Lausanne, Switzerland.

[2]Environmental Remote Sensing Laboratory (LTE), School of Architecture, Civil & Environmental Engineering, Ecole Polytechnique Fédérale de Lausanne, Lausanne, Switzerland

[3]Laser Remote Sensing Unit (LRSU), Physics Department, National Technical University of Athens, Zografou, Greece

[4]Center for Studies of Air Quality and Climate Change, Institute of Chemical Engineering Sciences, Foundation for Research and Technology Hellas, Patras, Greece.

[5]ERL, Institute of Nuclear and Radiological Sciences and Technology, Energy and Safety, National Centre of Scientific Research “Demokritos”, Ag. Paraskevi, Greece

[6]Division of Environmental Physics and Meteorology, Department of Physics, University of Athens, Athens, Greece

[*]Corresponding author: Athanasios Nenes (athanasios.nenes@epfl.ch)

Supplementary Tables

Supplementary Table 1

W-band spectral zenith profiler (WProf) properties and parameters. WProf uses three chirps, whose ranges are: chirp 0: 104-1,245 m, chirp 1: 1,267-4,191 m, chirp 2: 4,221-9,981 m.

Frequency (GHz)	94
Transmission	Frequency-modulated continuous wave (FMCW)
3-dB beam width (°)	0.53
Time resolution (s)	5
Range resolution (m)	7.5 / 16 / 32
Nyquist velocity (m s^{-1})	10.8 / 6.92 / 3.3

Supplementary Texts

Supplementary Text 1: Evaluation of INP concentrations and their effects on the cloud structure

The default temperature-dependent scheme of the Weather Research and Forecasting (WRF) model shows a gradual increase in simulated ice crystal number concentrations (ICNCs), reaching peak concentrations exceeding 100 L^{-1} at temperatures below -30°C (Fig. 2a). However, when replacing the default primary ice production parameterizations with the aerosol-aware scheme utilized in the DEMOTT simulation, the colder-temperature ICNCs are reduced, with predicted values consistently remaining below 50 L^{-1} (Fig. 2b). It is worth noting here that the aerosol information used to constrain the Demott[1] parameterization was derived from the altitude of Helmos Hellenic Atmospheric Aerosol and Climate Change (HAC)[2], which is ∼500 m higher than the "Vathia Lakka" (VL) station. Although this may lead to slight underestimations of the ice nucleating particles (INPs) at lower altitudes and, conversely, an INP overestimation at altitudes above (HAC)[2], DEMOTT still predicts much lower ICNCs than CONTROL at cold temperatures and provides a more realistic representation of the upper-level INPs. Reducing the predicted ICNCs increases the liquid water content (LWC) present at temperatures below -20°C (Fig. 3b), which is non-existent for CONTROL (Fig. 3a). During the 3rd cloud period, in the absence of seeding ice particles from above, higher ICNCs predicted by CONTROL lead to effective growth through riming and WBF, allowing for differential settling and justifying the enhanced aggregation rates compared to DEMOTT (Figs. 2a, b).

Supplementary Text 2: Modeling uncertainties during the low-level orographic cloud period

In the orographic cloud persisting after the passage of the seeder cloud (3rd turquoise box in Fig. 1a), notable spikes of enhanced radar equivalent reflectivity factor (Ze_w) are observed, which are not fully captured by the simulation that accounts for ice multiplication, referred to as ALLSIP (Fig. 1d), but are comparatively better reproduced by the CONTROL and DEMOTT simulations (Figs. 1b, c). This is further supported by median profiles extracted from this period, wherein the predictions of the two simulations incorporating solely PIP parameterizations align more closely with the observations (Supplementary Fig. 4). The inefficiency of the ALLSIP simulation in replicating the observed Ze_w spikes might stem from uncertainties in the representation of implemented secondary ice production mechanisms within the bulk microphysics framework. For instance, Sotiropoulou[2] found that adopting an emulated bin framework for collisional break-up and droplet-shattering could enhance simulated ice multiplication rates, albeit with increased computational demand. Given the prevailing stormy conditions during the case study (Supplementary Fig. 2c), various surface-based processes such as blowing snow or detachment of surface hoar frost[3] could have potentially contributed to the

observed instances of enhanced Ze_w close to the surface. Simplified methods to simulate such processes have been explored for orographic MPCs[4,5], yet it would be intriguing to explore more sophisticated and advanced modeling frameworks[6,7]. Other than ice multiplication and surface-based processes, microphysical processes such as pre-activation of INPs[8,9], as demonstrated for example in Yang[10] for tropical maritime stratiform clouds, may also be important ICNC sources.

An additional plausible explanation might involve the underrepresentation of specific INP types. As shown by Gao[11], biological INPs play a crucial role, especially when the $(HAC)^2$ station is affected by the planetary boundary layer (PBL) or is located close to cloud top – conditions that are frequently met during the 3rd cloud period (see black circles in Supplementary Fig. 5). Biological INPs, active at relatively warm temperatures higher than -15°C, are indeed not considered in the DeMott[1] scheme.

Supplementary Text 3: Physics options employed in WRF

WRF is forced with initial and 6-hourly boundary conditions from the fifth generation of the European Centre for Medium-Range Weather Forecasts (ECMWF) atmospheric reanalyses dataset (ERA5)[12]. The use of high-resolution ERA5 data (0.36°) for initializing WRF has been found to outperform the near real-time Global Forecasting System (GFS) data from the National Centers for Environmental Protection (NCEP), which has a spatial resolution of 1° (not shown). The static fields at each model grid point, including topography and land use fields, were sourced from default WRF pre-processing system datasets with a resolution of 30''. The land use categories were based on the Moderate Resolution Imaging Spectroradiometer (MODIS) land cover classification. In terms of physics options, we employed the Rapid Radiative Transfer Model for General Circulation Models (RRTMG) radiation scheme to parameterize both shortwave and long-wave radiative transfer. The PBL physics were represented using the non-local, first-order closure YSU (Yonsei University) scheme[13], coupled with its associated surface layer scheme. A sensitivity simulation revealed that YSU outperformed the local Mellor-Yamada-Janjić (MYJ)[14] 1.5 order scheme (not shown) in terms of meteorological observations. Surface processes were modeled using the Noah land surface model (Noah LSM)[15]. The Kain-Fritsch cumulus parameterization was only activated in the 12-km resolution domain, as the resolution of the two nested domains was deemed sufficient to reasonably resolve cumulus cloud processes at the grid scale.

Supplementary Text 4: INP measurements at $(HAC)^2$

The Portable Ice Nucleation Experiment (PINE) is an innovative instrument designed for ice nucleation studies and long-term field observations of ice nucleating particles (INPs) across a wide temperature range. During the Cloud-AerosoL InteractionS in the Helmos background TropOsphere (CALISHTO) campaign, PINE was operated at the mountain-

top site of (HAC)[2]. PINE employs a pumped expansion principle to generate ice and water supersaturated conditions for testing the ice nucleation ability of aerosol particles. The instrument operates in repetitive cycles, involving the sampling of aerosol into a pre-cooled cloud chamber, activation of aerosol particles as supercooled droplets and ice crystals through the expansion of air inside the chamber, and subsequent refilling of the cloud chamber with fresh aerosol for the next cycle. A more detailed description of the PINE instrument can be found in Möhler[16]. The INP measurements presented in Complementary Fig. 10a correspond to the period spanning one month, from the end of October to the end of November 2021. A comprehensive analysis of INP sources during CALISHTO is provided in Gao[11].

Supplementary Figures

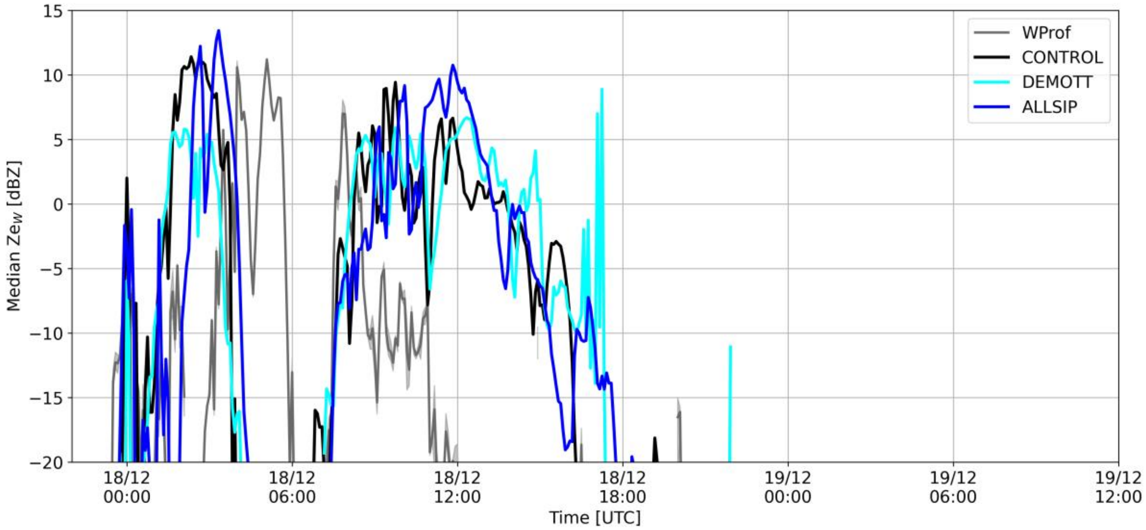

Supplementary Figure 1 Median timeseries of the radar equivalent reflectivity factor (Ze_w) calculated at an altitude ranging from 1.8 to ~2 km in the atmosphere. Ze_w is derived either from the WProf radar measurements (depicted by the grey line) collected at the VL station, or from simulations conducted by the CONTROL (black line), DEMOTT (cyan line), and ALLSIP (blue line) sensitivity simulations of WRF, coupled with the Cloud Resolving Model Radar Simulator (CR-SIM). In both the measured and simulated data, the presence of high reflectivity values (>-20 dBz) within this altitude range was used as a criterion for identifying each distinct cloud periods. This is because the first two cloud systems were associated with ice particles falling either from higher levels within the same cloud (internal seeding) or from an overlying cloud (external seeder-feeder), while the third one is a low-level orographic cloud.

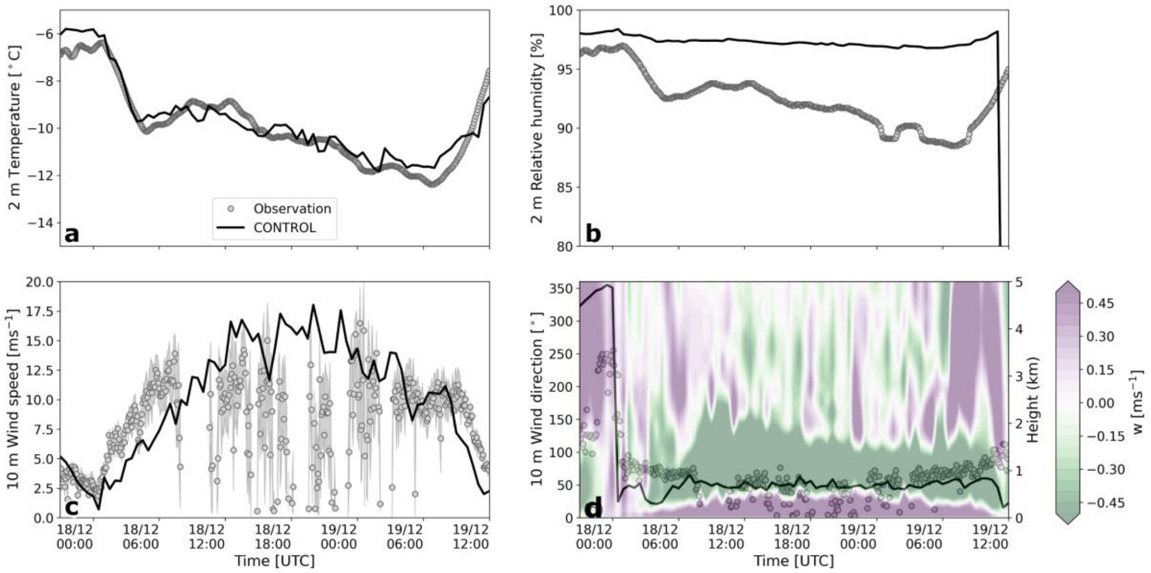

Supplementary Figure 2 Time series of (**a**) temperature and (**b**) relative humidity with respect to liquid phase at 2 m height, (**c**) wind speed and (**d**) wind direction at 10 m height. Gray circles indicate measurements collected between 17 and 19 December 2021 at the (HAC)^2 station, while modeled values from the CONTROL simulation of WRF are shown with a black line. The semi-transparent contour plot represents the vertical velocity (w) profile predicted by CONTROL.

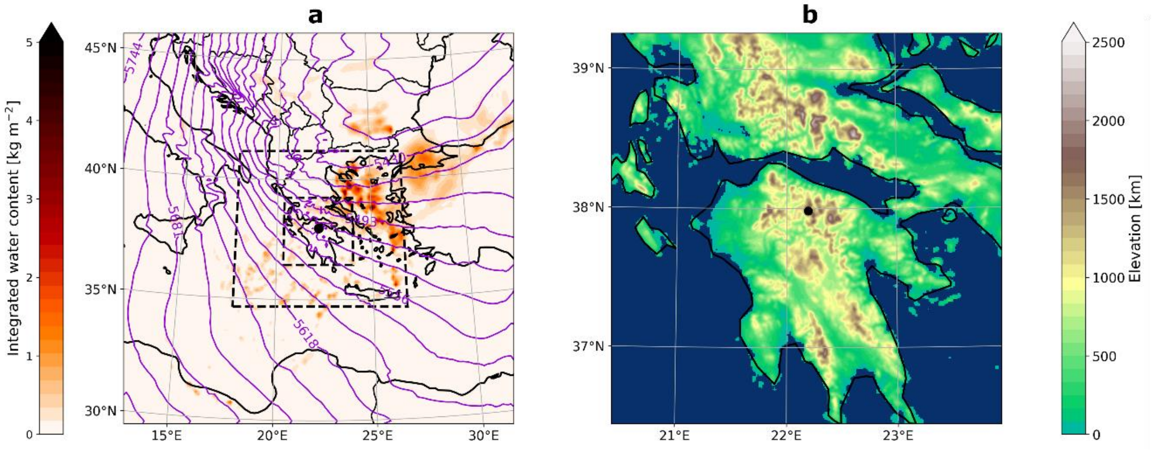

Supplementary Figure 3 (**a**) Vertically integrated water content obtained from the 12-km resolution domain of the CONTROL simulation of WRF on December 17 (22:00 UTC), 2021. The purple contours represent the 500 hPa geopotential height in meters. The black dashed lines outline the boundaries of the two nested domains of WRF. (**b**) Model orography from the 1-km resolution domain. The black dot in both panels marks the location of the $(HAC)^2$ station.

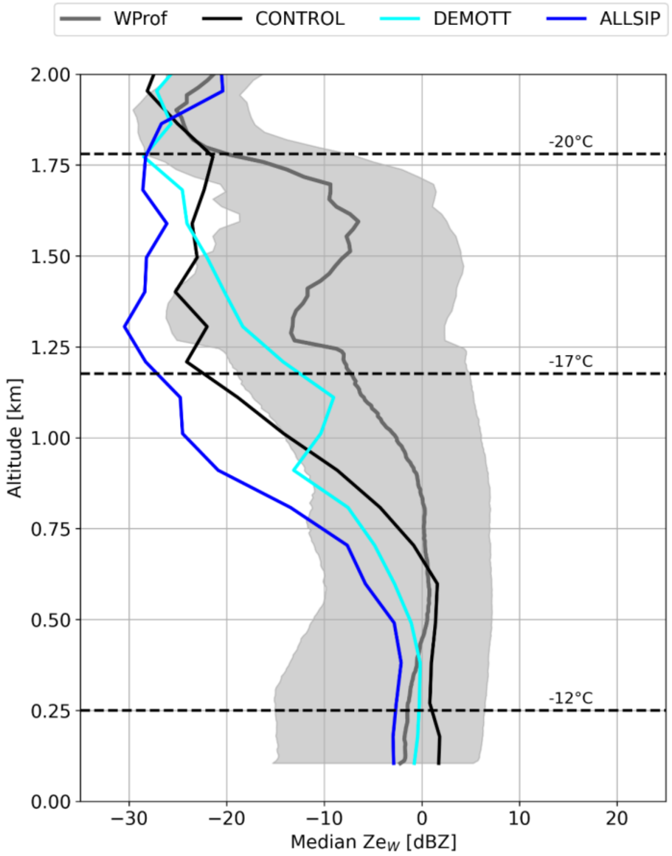

Supplementary Figure 4 Median time-height vertical profiles of observed and simulated radar reflectivity extracted during the low-level orographic cloud period. The grey line represents the median WProf observations, with the shaded region indicating the IQR. The black, cyan, and blue lines denote the results from the CONTROL, DEMOTT, and ALLSIP simulations, respectively. Temperature contours superimposed in these panels are from the ALLSIP simulation.

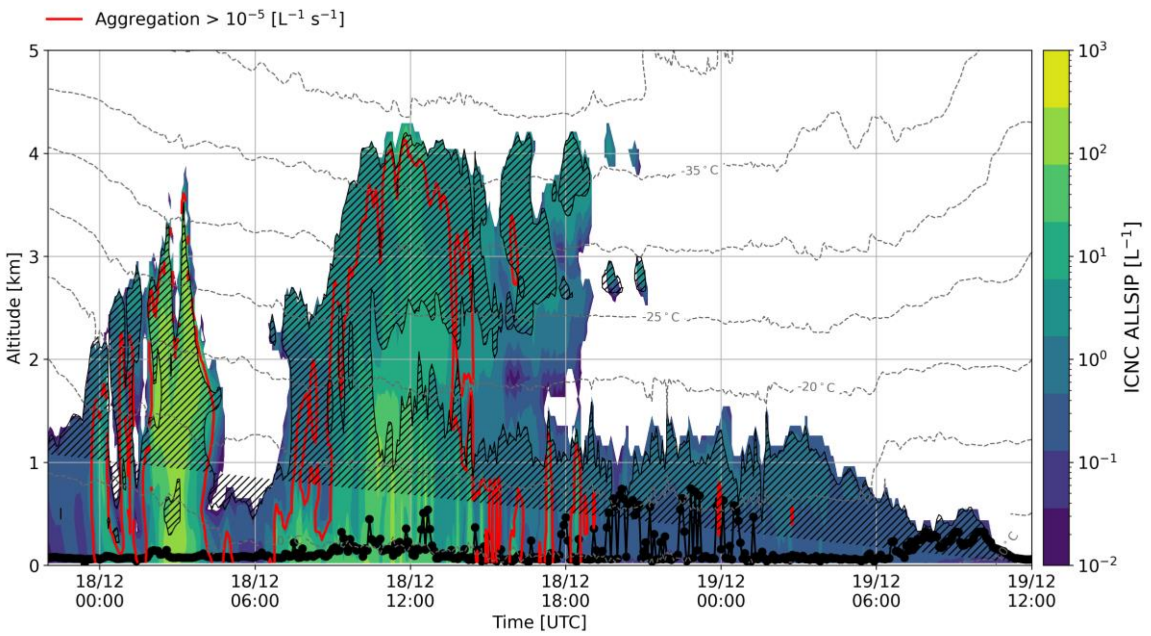

Supplementary Figure 5 Time-height plots of total ICNCs produced by the ALLSIP simulation, as in Figure 5c in the main manuscript. The grey contours represent temperature isotherms, while the red contours show areas where snowflake aggregation rates exceed 10^{-5} $L^{-1}s^{-1}$. The black hatched lines indicate regions that are supersaturated with respect to ice, while the black circle markers represent the PBL height relative to the VL station. Note that a PBL height exceeding 0.5 km implies that the high-altitude station of $(HAC)^2$ is in the PBL and directly influenced by local aerosol sources.

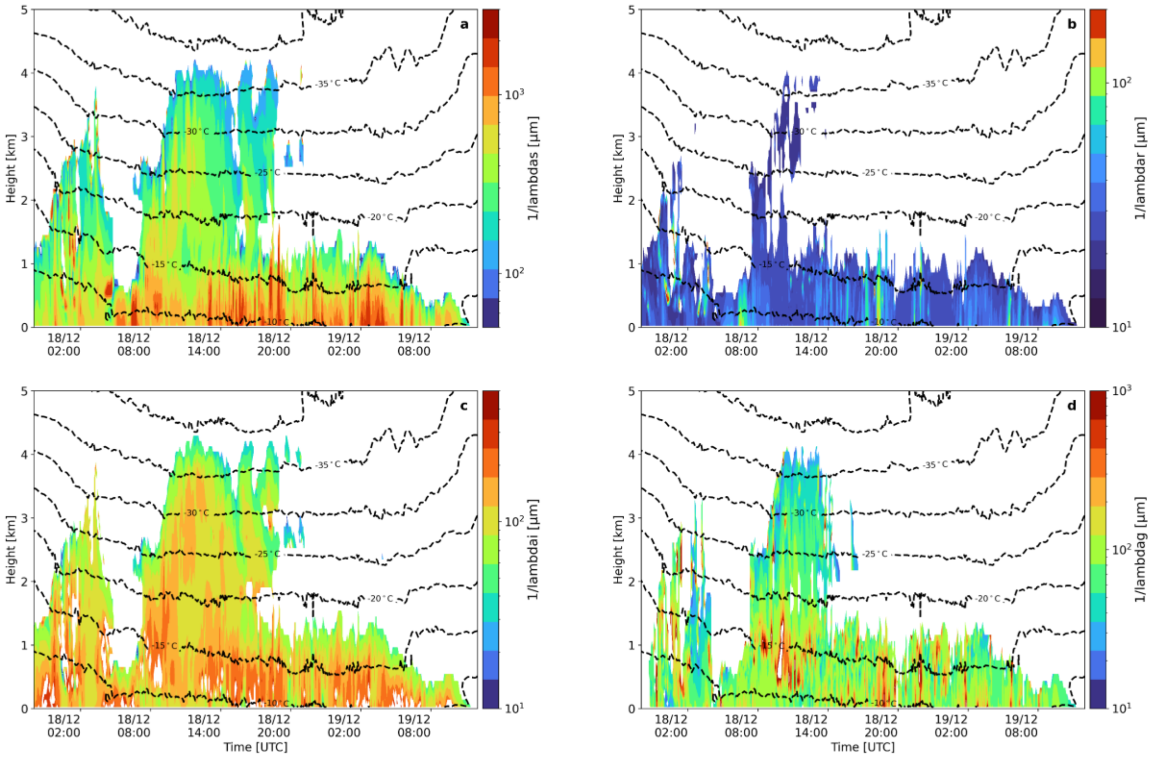

Supplementary Figure 6 Timeseries of characteristic sizes (in μm) inferred by reversing the slope parameter (lambda) that describes the exponential size distributions of (**a**) snow (1/lambdas), (**b**) raindrops (1/lambdar), (**c**) cloud ice (1/lambdai), and (**d**) graupel (1/lambdag) in the Morrison[17] microphysics scheme of WRF. These characteristic sizes are extracted from the ALLSIP sensitivity simulation of WRF, which demonstrates better agreement with radar observations.

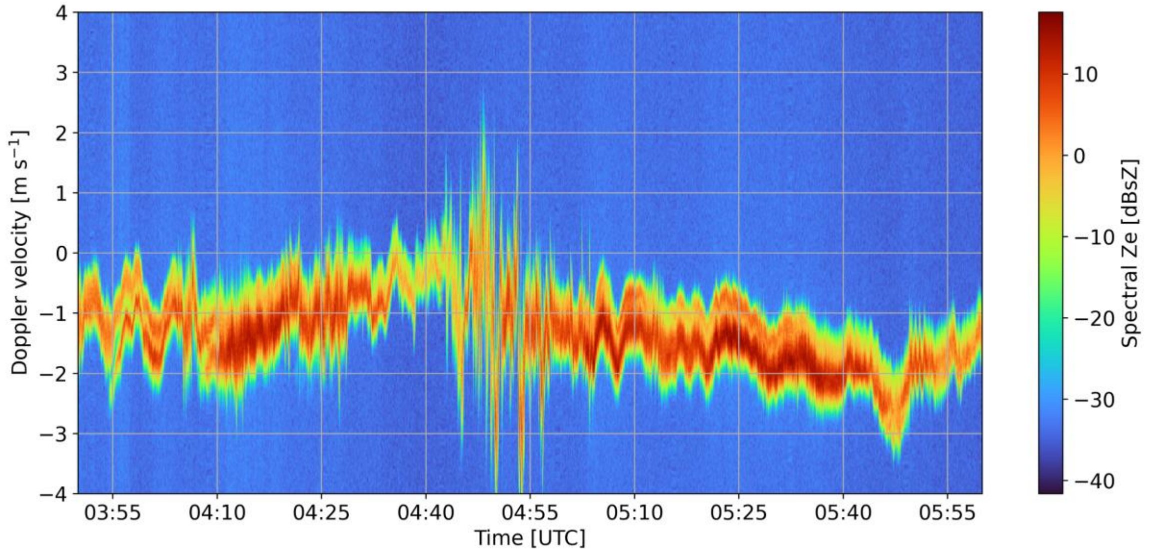

Supplementary Figure 7 Timeseries of WProf Doppler spectra extracted from an altitude of 1100 m above ground level, spanning the period between 03:50 UTC and 06:00 UTC on 18 December 2021, corresponding to the nimbostratus cloud period.

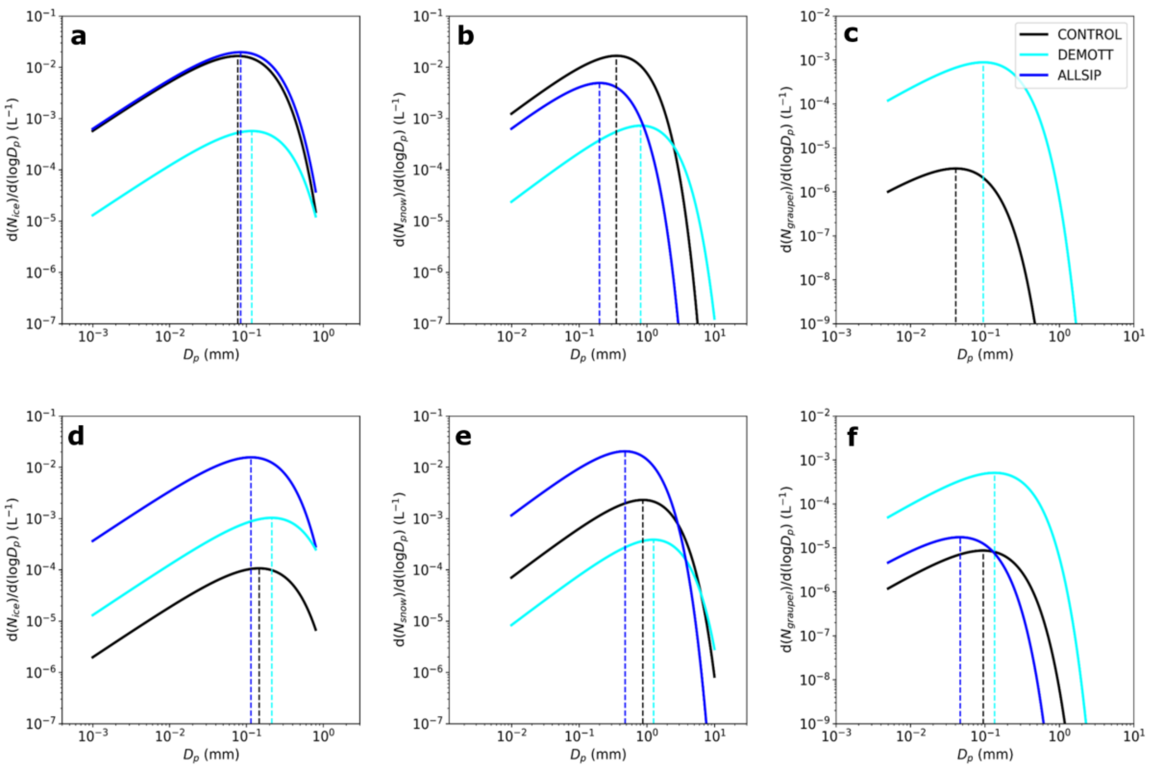

Supplementary Figure 8 Size distribution of (**a**, **d**) cloud ice, (**b**, **e**) snow and (**c**, **f**) graupel particles, predicted by CONTROL (black line), DEMOTT (cyan line) and ALLSIP (blue line) simulations of WRF. The size distributions were taken at 03:55 UTC on 18 December (nimbostratus cloud period) at 2.0 km (top panel) and at 0.7 km in the atmosphere (bottom panel). The vertical dashed lines indicate the mode of each distribution.

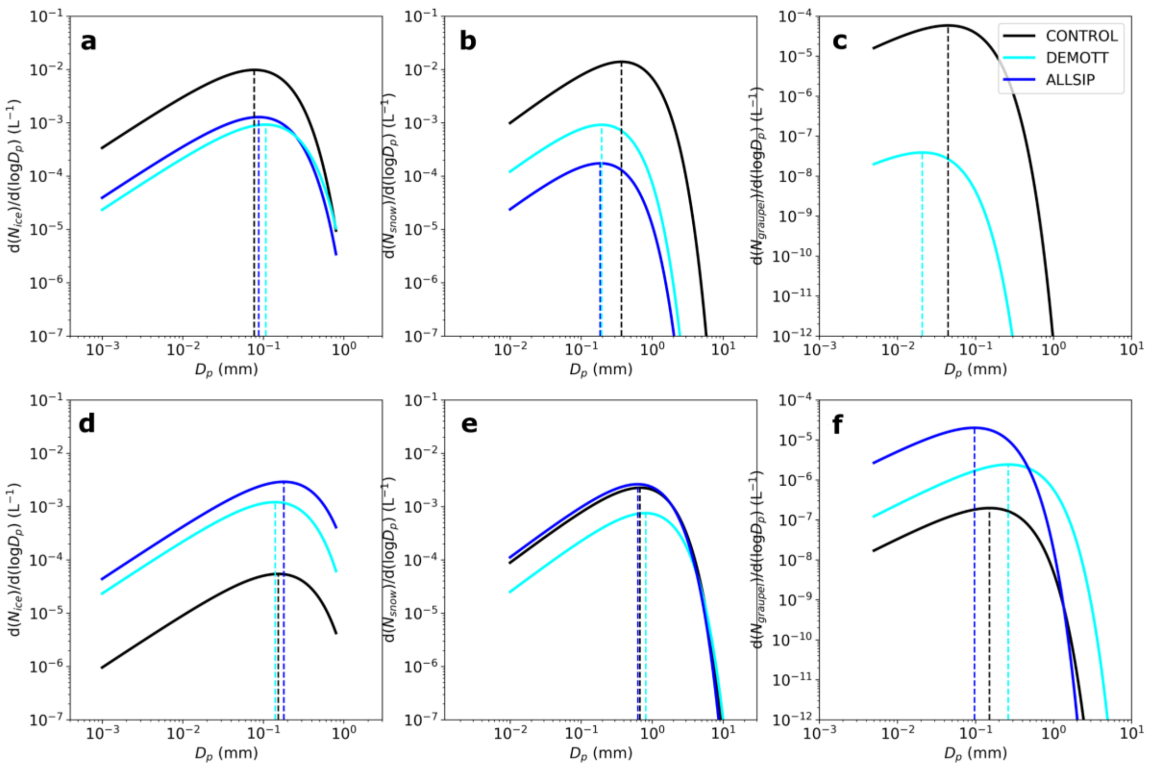

Supplementary Figure 9 Size distribution of (**a**, **d**) cloud ice, (**b**, **e**) snow and (**c**, **f**) graupel particles, predicted by CONTROL (black line), DEMOTT (cyan line) and ALLSIP (blue line) simulations of WRF. The size distributions were taken at 09:20 UTC on 18 December (seeder-feeder cloud period) at 2.2 km (top panel) and at 0.5 km in the atmosphere (bottom panel). The vertical dashed lines indicate the mode of each distribution.

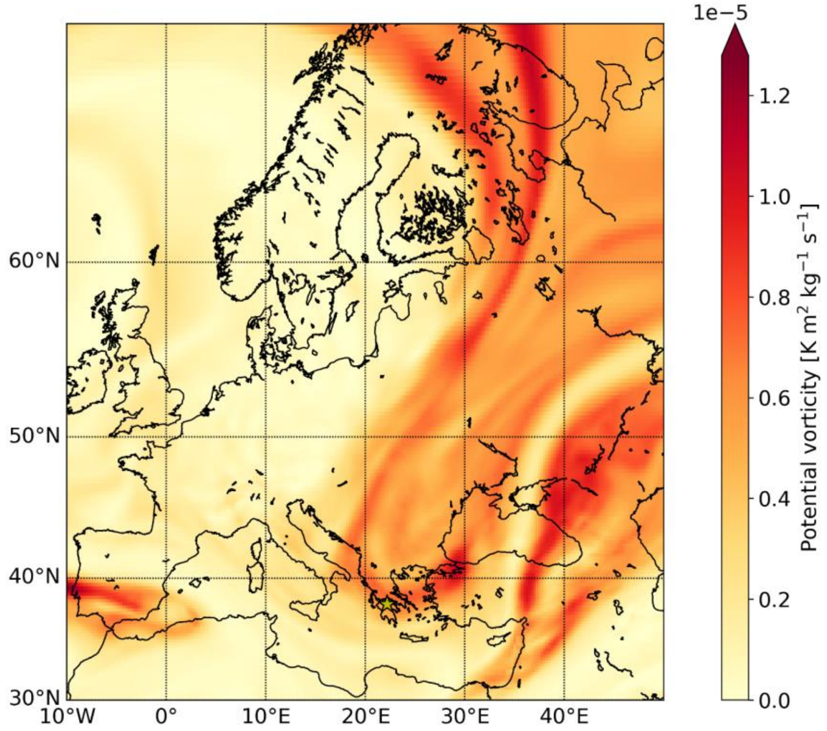

Supplementary Figure 10 Synoptic situation at 22:00 UTC on 17 December 2021 from ERA5 data. The color shading represents the potential vorticity (in K m^2 kg^{-1} s^{-1}) at the 320 K isentrope. The location of Mount Helmos is indicated by the yellow star.

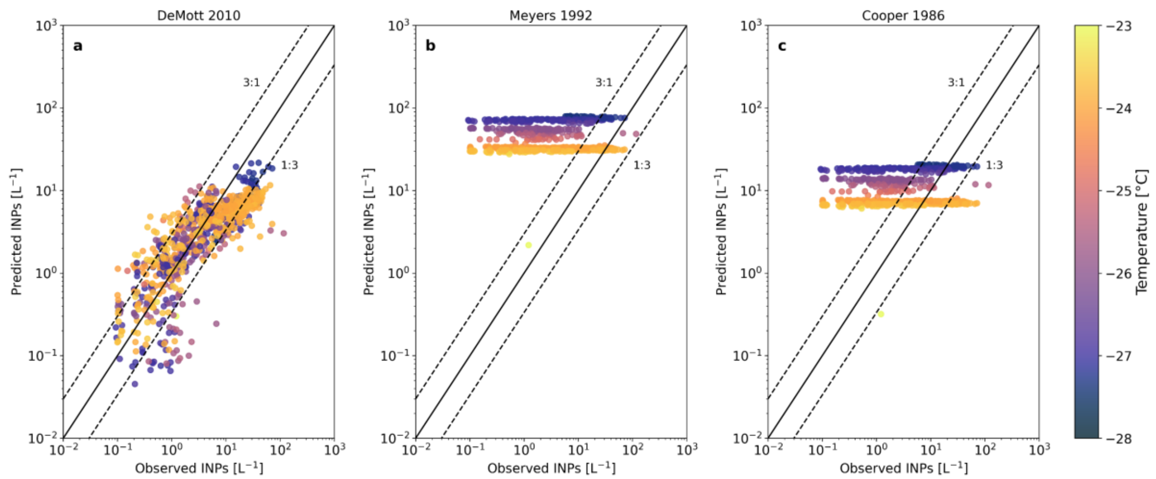

Supplementary Figure 11 Scatterplot of INPs predicted offline using the parameterized expressions developed by (**a**) DeMott[1], (**b**) Meyers[18], and (**c**) Cooper[19] vs the INPs measured at (HAC)[2] station by the PINE instrument. The INP measurements are color-shaded based on the PINE temperature (in °C).

Supplementary References

1. DeMott, P. J. *et al.* Predicting global atmospheric ice nuclei distributions and their impacts on climate. *Proc. Natl. Acad. Sci.* **107**, 11217–11222 (2010).

2. Sotiropoulou, G. *et al.* Sensitivity of Arctic clouds to ice microphysical processes in the NorESM2 climate model. *Prepr. Authorea* (2022) doi:10.1002/essoar.10512081.1.

3. Beck, A. *et al.* Impact of surface and near-surface processes on ice crystal concentrations measured at mountain-top research stations. *Atmos. Chem. Phys.* **18**, 8909–8927 (2018).

4. Georgakaki, P. *et al.* Secondary ice production processes in wintertime alpine mixed-phase clouds. *Atmos. Chem. Phys.* **22**, 1965–1988 (2022).

5. Farrington, R. J. *et al.* Comparing model and measured ice crystal concentrations in orographic clouds during the INUPIAQ campaign. *Atmos. Chem. Phys.* **16**, 4945–4966 (2016).

6. Gerber, F., Sharma, V. & Lehning, M. CRYOWRF—Model Evaluation and the Effect of Blowing Snow on the Antarctic Surface Mass Balance. *J. Geophys. Res. Atmos.* **128**, 1–19 (2023).

7. Sharma, V., Gerber, F. & Lehning, M. Introducing CRYOWRF v1.0: multiscale atmospheric flow simulations with advanced snow cover modelling. *Geosci. Model Dev.* **16**, 719–749 (2023).

8. Yang, J. *et al.* High ice concentration observed in tropical maritime stratiform mixed-phase clouds with top temperatures warmer than −8 °C. *Atmos. Res.* **233**, (2020).

9. Korolev, A. & Leisner, T. *Review of experimental studies of secondary ice production*. *Atmospheric Chemistry and Physics* vol. 20 (2020).

10. Jing, X. *et al.* Pre-Activation of Ice Nucleating Particles in Deposition Nucleation Mode: Evidence From Measurement Using a Static Vacuum Water Vapor Diffusion Chamber in Xinjiang, China. *Geophys. Res. Lett.* **49**, 1–9 (2022).

11. Gao, K. *et al.* Biological and dust aerosol as sources of ice nucleating particles in the Eastern Mediterranean: source apportionment, atmospheric processing and parameterization. Preprint at: https://doi.org/10.5194/egusphere-2024-511 (2024).

12. Hersbach, H. *et al.* The ERA5 global reanalysis. *Q. J. R. Meteorol. Soc.* **146**, 1999–2049 (2020).

13. Hong, S. Y., Noh, Y. & Dudhia, J. A new vertical diffusion package with an

explicit treatment of entrainment processes. *Mon. Weather Rev.* **134**, 2318–2341 (2006).

14. Janjić, Z. I. Nonsingular Implementation of the Mellor-Yamada Level 2.5 Scheme in the NCEP Meso model. *NOAA/NWS/NCEP Off. note 437* 61 pp (2002).

15. Chen, F. & Dudhia, J. Coupling and advanced land surface-hydrology model with the Penn State-NCAR MM5 modeling system. Part I: Model implementation and sensitivity. *Mon. Weather Rev.* **129**, 569–585 (2001).

16. Möhler, O. *et al.* The Portable Ice Nucleation Experiment (PINE): A new online instrument for laboratory studies and automated long-term field observations of ice-nucleating particles. *Atmos. Meas. Tech.* **14**, 1143–1166 (2021).

17. Morrison, H., Curry, J. A., Shupe, M. D. & Zuidema, P. A new double-moment microphysics scheme for application in cloud and climate models. Part II: Single-column modeling of Arctic clouds. *J. Atmos. Sci.* **62**, 1678–1693 (2005).

18. Meyers, M. P., DeMott, P. J. & Cotton, W. R. New Primary Ice-Nucleation Parameterizations in an Explicit Cloud Model. *J. Appl. Meteorol.* **31**, 708–721 (1992).

19. Cooper, W. A. Ice Initiation in Natural Clouds. *Meteorol. Monogr.* **21**, 29–32 (1986).
